# Evolutionary Origin of Insulin-Degrading Enzyme and Its Subcellular Localization and Secretion Mechanism: A Study in Microglial Cells

**DOI:** 10.3390/cells11020227

**Published:** 2022-01-11

**Authors:** Miriam Corraliza-Gómez, Concepción Lillo, Irene Cózar-Castellano, Eduardo Arranz, Diego Sanchez, Maria D. Ganfornina

**Affiliations:** 1 Instituto de Biología y Genética Molecular, Excellence Unit, University of Valladolid-CSIC, 47003 Valladolid, Spain; irene.cozar@uva.es (I.C.-C.); earranz@uva.es (E.A.); lazarill@ibgm.uva.es (D.S.); opabinia@ibgm.uva.es (M.D.G.); 2 Instituto de Neurociencias de Castilla y León (INCYL), University of Salamanca, 37007 Salamanca, Spain; conlillo@usal.es; 3 Hospital Virgen de la Vega-Instituto de Investigación Biomédica de Salamanca (IBSAL), 37007 Salamanca, Spain; 4 Centro de Investigación Biomédica en Red de Diabetes y Enfermedades Metabólicas Asociadas (CIBERDEM), 28029 Madrid, Spain

**Keywords:** insulin-degrading enzyme, phylogeny, molecular evolution, intron-exon structure, microglia, lipid rafts, extracellular vesicles, inflammatory state, amyloid β, oxidative stress

## Abstract

The insulin-degrading enzyme (IDE) is a zinc-dependent metalloendopeptidase that belongs to the M16A metalloprotease family. IDE is markedly expressed in the brain, where it is particularly relevant due to its in vitro amyloid beta (Aβ)-degrading activity. The subcellular localization of IDE, a paramount aspect to understand how this enzyme can perform its proteolytic functions in vivo, remains highly controversial. In this work, we addressed IDE subcellular localization from an evolutionary perspective. Phylogenetic analyses based on protein sequence and gene and protein structure were performed. An in silico analysis of IDE signal peptide suggests an evolutionary shift in IDE exportation at the prokaryote/eukaryote divide. Subcellular localization experiments in microglia revealed that IDE is mostly cytosolic. Furthermore, IDE associates to membranes by their cytoplasmatic side and further partitions between raft and non-raft domains. When stimulated, microglia change into a secretory active state, produces numerous multivesicular bodies and IDE associates with their membranes. The subsequent inward budding of such membranes internalizes IDE in intraluminal vesicles, which later allows IDE to be exported outside the cells in small extracellular vesicles. We further demonstrate that such an IDE exportation mechanism is regulated by stimuli relevant for microglia in physiological conditions and upon aging and neurodegeneration.

## 1. Introduction

The insulin-degrading enzyme (IDE; EC 3.4.24.56, also known as insulysin, insulin protease or insulinase) was discovered more than 70 years ago and was named after its ability to strongly bind and degrade insulin in tissue extracts [1]. IDE is a 110 kDa zinc-metalloendopeptidase that belongs to the clan ME family M16A of metalloproteases, also known as “inverzincins” because they are characterized by a zinc-binding consensus motif (HxxEH, where “x” means any amino acid) that is inverted with regard to the sequence in the majority of conventional M16 peptidases (HExxH) [2,3]. Metalloendopeptidases of the ME clan are ubiquitously present throughout the tree of life and include proteases with highly divergent primary sequences but a strikingly high 3D structure conservation [4].

IDE is ubiquitously expressed, with its highest expression in the testes, liver, muscle and brain [5,6]. The omnipresence of IDE in different cell types, blood [7] and even in cerebrospinal fluid [8] suggests that this ubiquitous protein performs crucial functions in many different tissues. The discovery of IDE as an insulin-degrading enzyme in 1949 [1] and as an Aβ-degrading enzyme in 1994 [9] raised questions about the precise location of IDE interaction with these substrates, which are secreted to the extracellular compartment. Since then, IDE subcellular localization has been extensively studied; however, it remains controversial. IDE was found mainly in the cytosolic compartment [10,11], but it has been reported in many other subcellular compartments, including mitochondria [12], endosomes [13,14,15], peroxisomes [16], multivesicular bodies [17,18], plasma membrane [19,20,21], on the cell surface [22,23,24], at the extracellular space [8] and in exosomes [17,18,25]. These contradictory antecedents reflect the need to address IDE subcellular localization to understand how it can perform its proposed functions.

In this work, we first address this crucial cell biology question from an evolutionary perspective. An amino acid-based phylogeny of selected representatives helped us to contextualize IDE in the clan ME gene superfamily. We then carried out a global search of IDE relatives, recovered proteins of the M16A family and studied their phylogenetic relationships based on protein sequence alignments, gene architecture and protein tertiary structure. We used the phylogenetic tree of IDE homologs to explore the evolution of their predicted signal peptide and subcellular localization. Our study suggests a shift in exportation between prokaryotic and eukaryotic cells. Our in silico studies led us to experimentally assess IDE localization in microglial cells, and we found IDE mostly in the cytoplasm and distributed between soluble and membrane fractions. IDE association to membranes only occurs at their cytosolic side, and membrane-associated IDE further partitions between raft and non-raft fractions. Extracellular vesicles arisen from multivesicular bodies mediate IDE secretion, and such an IDE export mechanism is dependent on the activation state of microglial cells, and is also affected by stimuli, such as oxidative stress and exposure to Aβ oligomers.

## 2. Materials and Methods

### 2.1. Bioinformatic Analyses

#### 2.1.1. Sequences Selected for Clan ME Analysis

Proteins from clan ME were selected according to the “Handbook of proteolytic enzymes” [4]. The proteins analyzed are summarized in Table 1.

#### 2.1.2. Searching for IDE Homologous Sequences

Our initial question was to find protein sequences homologous to IDE in any life kingdom. The Position-Specific Iterated–Basic Local Alignment Search Tool (PSI-BLAST) algorithm [26] was used to perform a sequence search to find homologous proteins to human IDE. The search was restricted to the non-redundant protein sequence global database, excluding models and environmental samples, using a low-complexity region filter, the BLOSUM62 substitution matrix and E-value ≤ 1 × 10^−15^. General selection criteria were a protein sequence identity ≥25% and coverage ≥50%. After this initial search, 205 protein sequences were selected to build a PSSM (Position-Specific Scoring Matrix, see Supplementary Materials File S1) to maximize the retrieval of IDE distant homologues. Subsequent BLAST searches were performed individually in all taxonomic groups using the IDE PSSM and maintaining the same criteria described above. This search protocol allowed us to retrieve all M16A family members with four domains, which were compiled in an IDE-related database (Supplementary Materials File S3—Tables S1–S4). To avoid overloading with redundant sequences (e.g., truncated proteins identical to their longer form), sequences belonging to the same organism and showing ≥99% identity were excluded.

#### 2.1.3. Multiple Sequence Alignments (MSA)

Selected M16A sequences were aligned using the MAFFT (Multiple Alignment using Fast Fourier Transform) online server [27], according to the following workflow: (1) to compare primary sequences from ME proteins, we used the MAFFT-L-ins-i algorithm. (2) To study the global phylogeny of all IDE homologous proteins, we first built an MSA from *Chordata* using the iterative algorithm L-ins-i. Then, we used the MAFFT-Add algorithm to include the remaining sequences with a progressive L-ins-i method, keeping alignment length (parameters: BLOSUM62; Gap 1.53; Offset 0.0). (3) To study the evolution of IDE across species, sequences from 14 representative organisms were selected and aligned using the MAFFT E-ins-i algorithm, including a structural alignment between human IDE (PDB Id: 3CWW) and pitrilysin (1Q2L). (4) To analyze the evolution of IDE and nardilysin (NRD) individually in *Chordata*, we selected 8 representative organisms and generated MSAs using the progressive G-ins algorithm. (5) To compare IDE and NRD gene structures, we selected 13 vertebrates that express both proteins and performed an MSA of all the sequences using the iterative algorithm E-ins-i. MSAs were visualized with Alignment Viewer (http://alignmentviewer.org).

#### 2.1.4. Phylogenetic Reconstruction

Phylogenetic trees based on MSAs were inferred using the IQ-TREE online server (http://iqtree.cibiv.univie.ac.at). First, a best-fit substitution model was selected by running the ModelFinder algorithm [28]. Then, a maximum likelihood (ML) tree was created by using the algorithm implemented in the IQ-TREE server [29]. Finally, branch support analyses were performed with an Ultrafast Bootstrap approximation (1000 replicas) [27]. The resultant phylogenetic tree was visualized using FigTree v1.4.4 (http://tree.bio.ed.ac.uk/software/figtree/).

#### 2.1.5. Intronic Features Extraction

To compare IDE and NRD gene structures, 13 vertebrate taxa were selected. From protein sequences, an automatic search was carried out for the corresponding gene on the WebScipio online server [30]. For each species, the genomic assembly with the best N50 value was selected. Thus, 26 gene structures were retrieved. The MSA and gene structure of the 26 protein sequences were used by the GenePainter online server [31] to map the intron phase (0, 1 or 2) and position in the protein MSA.

#### 2.1.6. Protein Structure Alignments

Protein structures were collected from the Protein Data Bank (PDB) server. Highly accurate structure predictions from proteins not available at PDB were retrieved from either the AlphaFold database [32] or the SwissProt 3D website. Protein structures were aligned using the Dali server [33], and a correspondence analysis was performed on the same server [34] to visualize the structural relationships of the protein set in a two-dimension plot.

#### 2.1.7. Bioinformatic Prediction of Subcellular Localization

The presence of signal peptides in IDE homologs was predicted using the SignalP5.0 server [35]. The subcellular localization of M16A eukaryotic proteins was predicted using the DeepLoc-1.0 server [36].

### 2.2. Animals, Cell Cultures and Treatments

The IDE-knockout (IDE-KO) mouse (Ide^tm1a(EUCOMM)Wtsi^, Mouse Genome Informatics identifier MGI:4431946 [37]), kindly provided by Dr. M.A. Leissring (University of California, Irvine, CA, USA), was backcrossed with wildtype (WT) mice on a C57BL/6J background (Charles River, Ecully, France), and the colony was maintained by crossing heterozygous animals. Mice were maintained in positive pressure-ventilated racks at 25 ± 1 °C with a 12 h light/dark cycle, fed ad libitum with standard rodent pellet diet (Harlan Inc., Indianapolis, IN, USA) and allowed free access to filtered and UV-irradiated water. Experimental procedures were approved by the University of Valladolid Animal Care and Use Committee, following the regulations of the Care and the Use of Mammals in Research (European Commission Directive 86/609/CEE, Spanish Royal Decree ECC/566/2015). Mouse genotypes were evaluated by PCR (Supplementary Materials File S2—Figure S1). To detect IDE-KO alleles, two parallel amplification reactions (35 cycles (30 s at 94 °C, 30 s 62 °C, 45 s 72 °C) plus 15 min at 72 °C final extension) were performed: one targeting exon 3 (Ex-F, 5′-TTCCTGTGCCCCTTGTTTGA-3′ and Ex-R, 5′-GTACGTTTGAAGCCCCGGTA-3′) and the other targeting one intronic region (Int-F, 5′-AACTGCCACCTGTCCAATCC-3′ and Int-R 5′-GGAAACCACTATGCCTACCTCT-3′). An example of genotyping results is shown in Supplementary Materials File S2—Figure S1. 

Primary microglial cultures were prepared from individual postnatal day 0 (P0) mice, following the method described by Saura et al. [38] with minor modifications. Briefly, cerebral cortices were immersed in dissection media (BSA 3.2 μg/mL DNaseI 27.5 μg/mL, penicillin 100 U/mL, streptomycin 100 U/mL, amphotericin B 0.25 μg/mL in EBSS medium) and minced with a surgical blade. Tissue was then pelleted by centrifugation (200× *g*, 2 min), resuspended in digestion media (BSA 3 μg/mL, DNaseI 60 μg/mL, penicilin 100 U/mL, streptomycin 100 U/mL, amphotericin B 0.25 μg/mL, trypsin 0.25 mg/mL in EBSS) and incubated for 15 min at 37 °C under agitation. Digested tissue was centrifuged (200× *g*, 5 min) and mechanically dissociated with a Pasteur pipette. Finally, individual cells were plated onto a 75 cm^2^-flask and incubated at 37 °C in 5% CO_2_ with 95% humidity to produce a mixed glial culture. Culture medium (DMEM/F-12 supplemented with 10% heat-inactivated fetal bovine serum (FBS), 2 mM L-glutamine, 100 U/mL penicillin and 100 U/mL streptomycin) was replaced one day after and then every 5–7 days. After 20 days in vitro, culture medium was replaced, and, 24 h later, the astrocytic upper layer was detached by mild trypsinization (0.8 mg/mL Trypsin-EDTA in DMEM/F-12, for 1 h at 37 °C). The remaining microglial cells were washed with PBS and incubated in conditioned medium from the mixed glial culture supplemented with macrophage colony stimulator factor (M-CSF, 25 ng/mL). In order to obtain “resting” microglia, isolated microglia remained for 72 h in these conditions before being used in any experiment.

The murine microglial BV-2 cell line was obtained from ATCC (American Type Culture Collection, Manassas, VA, USA). Cells were grown at 37 °C in a humidity-saturated atmosphere containing 5% CO_2_. Culture medium (RPMI 1640 Medium supplemented with 5% heat-inactivated FBS, 2 mM L-glutamine, 100 U/mL penicillin and 100 U/mL streptomycin) was replaced twice a week, and cells were sub-cultured at 50% confluence.

Cell treatments were performed in serum-free RPMI medium for 24 h. The stimuli used were: 100 ng/mL LPS (lipopolysaccharide from *E. coli* 0111:B4, Sigma-Aldrich, St. Louis, MO, USA); 25 μM paraquat (PQ; Sigma-Aldrich, St. Louis, MO, USA); 20 ng/mL IL-4 + 50 ng/mL IL-13 (PeproTech, Hamburg, Germany); and 1 μM Aβ oligomers (Bachem, Bubendorf, Switzerland). Aβ oligomers were prepared as described by Nuñez et al. [39]. After treatments, cells were collected to analyze membranes and intracellular proteins, while extracellular media were cleaned from detached cells and debris (as indicated below) and stored at −80 °C until processed.

### 2.3. Crude Membrane Preparations

Cells were grown in 75 cm^2^ flasks up to 80% confluence, and then harvested with GHCKS (2 g/L D-glucose, 4.77 g/L HEPES, 6.37 g/L NaCl, 0.3 g/L KCl, 3 g/L Na_3_Citrate·2H_2_O, pH 7.5) solution for BV-2 cells or mild trypsin (1.25 mg/mL) for primary glial cultures. Samples were centrifuged (300× *g*, 5 min). After complete removal of the supernatant, cell pellets were frozen until further processing.

Cell pellets were mechanically homogenized in TNE-PI [50 mM Tris-HCl pH 7.4, 150 mM NaCl, 5 mM EDTA, supplemented with protease inhibitors (PI; Roche, Basel, Switzerland)] in a Dounce homogenizer (Sartorius, Göttingen, Germany) with 20 strokes at 400 rpm on ice. Then, samples were low-speed centrifuged (3000× *g*, 10 min, 4 °C), and two phases were obtained: a pellet composed of dense cellular elements (nuclei, endoplasmic reticulum, mitochondria, etc.) and a soluble supernatant. The pellet was resuspended in lysis buffer (10 mM HEPES pH 7.6, 100 mM KCl, 1 mM EDTA pH 8.0, 0.5% sodium deoxycholate, 1% NP-40, 0.1% SDS, 10% glycerol, 1 mM DTT) supplemented with PI, while the supernatant was transferred into a Quick-Seal tube and ultracentrifuged (100 Ti rotor, 100,000× *g*, 75 min, 4 °C) in a Beckman Coulter Optimal-100XP Ultracentrifuge. After centrifugation, two phases were obtained: supernatant, composed by cytosolic content, and pellet, constituted by cell membranes. The membrane pellet was resuspended in lysis buffer, while the supernatant was concentrated using Amicon Ultra-4 Centrifugal Filter Devices (Merck-Millipore, Darmstadt, Germany) following the manufacturer’s instructions. Protein concentrations were quantified using the Micro BCA protein assay kit (Thermo Scientific, Waltham, MA, USA).

### 2.4. Isolation of Lipid Rafts by Sucrose Gradient Centrifugation

Membrane preparations were performed as described above, but the membrane pellets were resuspended in TNE-PI. Three different methods of lipid raft isolation were performed:

Triton-X100: The protein/detergent ratio is a critical parameter for correct Triton-X100-resistant membranes isolation [40]. This parameter was empirically determined for the BV-2 cell line as 400 μg proteins/μL Triton-X100. Particularly, 2 mg of membrane proteins were incubated in 0.5% (*v/v*) Triton-X100 in TNE-PI for 30 min at 4 °C in orbital agitation.

Triton-X114: 400 μg of membrane proteins were incubated in 1% (*v/v*) Triton-X114 in TNE-PI for 40 min at 4 °C in orbital agitation.

Sonication: 750 μg of membrane proteins were subjected to 5 sonication pulses using a probe sonicator (Vibra-Cell Bioblock scientific ultrasonic processor, Sonics & Materials, Newtown, CT, USA). Each pulse (20 s, 30% amplitude) was followed by 1 min incubation on ice.

After the corresponding membrane preparation treatment (detergent or sonication), the sample was immediately transferred to an Ultra-Clear centrifuge tube containing 2.25 mL of 80% (*w*/*v*) sucrose solution in TNE-PI to have the sample in a final concentration of 55% sucrose (lowest part of the gradient). A discontinuous sucrose gradient was created by adding, very gently, 6 mL of 35% sucrose in TNE-PI and then 3 mL of 5% sucrose in TNE-PI. The gradients were ultracentrifuged (SW40 rotor, 100,000× *g*, 21 h, 4 °C), and a total of twelve 1 mL fractions were collected from top to bottom. Each gradient fraction was incubated in 20% (*w*/*v*) trichloro-acetic acid (TCA) for 20 min. Protein precipitates were washed twice with cold ethanol, centrifuged (16,000× *g*, 30 min, 4 °C) and lyophilized in a SpeedVac centrifuge (800× *g*, 15 min, 35 °C). Dried protein samples were resuspended in protein sample buffer (63 mM Tris-HCl pH 6.8, 10% glycerol, 2% SDS, 100 mM DTT, 0.05% bromophenol blue) for subsequent discontinuous SDS-PAGE.

### 2.5. Immunoblot Analysis

Conditioned media or membrane and sucrose gradient fractions were analyzed by immunoblot under denaturing and reducing conditions (0.5% SDS, 25 mM DTT). Proteins were transferred to PVDF membranes (Immobilon-P, Merck-Millipore, Darmstadt, Germany) using standard procedures and exposed to rabbit serum anti-IDE (1:40,000; #AB9210, Merck-Millipore, Darmstadt, Germany), mouse anti-PMCA (1:1000 (0.2 μg/mL); Santa Cruz Biotechnology, Dallas, TX, USA), mouse anti-flotillin 1 (1:1000 (0.45 μg/mL); Becton Dickinson, Madrid, Spain), rabbit serum anti-CD81 (1:1000 (1 μg/mL); GeneTex, Alton Pkwy Irvine, CA, USA), anti-β-actin-HRP (1:200,000 (0.015 μg/mL); Sigma-Aldrich, St. Louis, MO, USA) and followed by HRP-conjugated secondary antibodies (1:10,000; Jackson ImmunoResearch, West Grove, PA, USA). Membranes were developed with enhanced chemiluminescence reagents (ECL; Merck-Millipore, Darmstadt, Germany), and the signal was visualized with a digital camera (VersaDoc; BioRad, Madrid, Spain). The integrated optical density of the immunoreactive protein bands was measured in images taken within the linear range of the camera, avoiding signal saturation and using the Quantity One 1-D Analysis Software (BioRad, Madrid, Spain).

### 2.6. Immunocytochemistry

Post-fixation labeling: BV-2 cells attached to 12 mm diameter glass coverslips were fixed with 2% phosphate-buffered formaldehyde (Polysciences, Heidelberg, Germany) for 10 min at room temperature (RT). Following washes in PBS, cells were blocked with 1% normal goat serum (NGS) either in PBS (non-permeabilizing conditions) or in 0.1% (*v/v*) Tween-20 in PBS (permeabilizing conditions). Cells were then incubated with primary antibodies [rabbit serum anti-IDE (1:10,000; #AB9210, Merck-Millipore, Darmstadt, Germany) and rat anti-CD11b (1:300; DSHB, Iowa Ave, IA, USA)] in blocking solution overnight at 4 °C. Alexa Fluor 488 or 594-conjugated IgGs (Jackson ImmunoResearch, West Grove, PA, USA) were used as secondary antibodies (1:2000 (0.75 μg/mL)). After washes in PBS, samples were mounted with Vectashield Vibrance with DAPI (Vector Laboratories, Burlingame, CA, USA). Starting with the initial settings described by Fernandez-Diaz et al. [41], IDE immunolabeling conditions were validated in our samples following antibody dilution series (primary antibody from 1:2000 to 1:40,000; secondary antibody from 1:1000 to 1:2000), tests of different fixatives (2–4% formaldehyde, ice-cold methanol) and blocking/permeabilizing conditions (1% NGS, 1% BSA, 0.1% Tween-20 or Triton-X100). A final validation of our protocol was performed by testing with WT and IDE-KO microglial cells (Supplementary Materials File S2—Figure S4).

Direct labeling of live cells in culture: live BV-2 cells attached to 12 mm diameter glass coverslips were incubated at 4 °C (5 min) to stop the endocytosis machinery. Then, cells were exposed to a cocktail of antibodies (rabbit anti-IDE 1:5000 plus rat anti-CD11b 1:300 in RPMI medium with 1% FBS) for 20 min at RT. After washes with PBS, cells were fixed with 4% formaldehyde in PBS for 10 min at RT. Then, cells were permeabilized with 1% NGS in 0.1% (*v*/*v*) Tween-20 in PBS. Alexa Fluor 488 and 594-conjugated IgGs (Jackson ImmunoResearch, West Grove, PA, USA) were used as secondary antibodies (1:2000 (0.75 μg/mL)). After washes in PBS, samples were mounted with Vectashield Vibrance with DAPI (Vector Laboratories, Burlingame, CA, USA).

Labeled cells were visualized with an Eclipse 90i fluorescence microscope (Nikon, Amsterdam, The Netherlands) equipped with a DS-Ri1 (Nikon, Amsterdam, The Netherlands) digital CCD (charge-coupled device) camera. Confocal images were obtained with a 63X oil immersion objective (HCX PL Apo CS NA = 1.4; Leica Microsystems, Wetzlar, Germany) attached to a confocal DMI 6000B microscope with a TCS SP8 confocal system (Leica Microsystems, Wetzlar, Germany) equipped with AOBS and AOTF systems. Fluorophores were excited with a WLL laser and a 405 line controlled by LAS AF software (Leica Microsystems, Wetzlar, Germany).

Images were acquired under the same conditions of illumination, diaphragm and condenser adjustments, exposure time, background correction and color levels. Images were analyzed using ImageJ software (National Institutes of Health, Bethesda, MD, USA).

### 2.7. Electron Microscopy

After validating with fluorescence microscopy, the fixation and blocking conditions stated below, BV-2 cells were plated on 12 mm diameter plastic coverslips at a density of 175,000 cells/cm^2^. One day after plating, cells were cultured in a low-serum medium (1% FBS-Charcoal stripped) for 24 h. Then, cells were fixed in 4% paraformaldehyde plus 0.3% glutaraldehyde in PBS for 10 min at RT under agitation. Following washes in PBS, cells were blocked with 1% NGS in PBS-Tween-20 0.1% (*v*/*v*) for 30 min at RT under agitation. Microglial cells were then incubated with rabbit serum anti-IDE primary antibody (1:10,000) in blocking solution overnight at 4 °C and washed three times with PBS. Samples were later incubated with ultra-small gold-conjugated goat anti-rabbit secondary antibodies (EMS, Hatfield, PA, USA) in PBS for 48 h at 4 °C. After several washes with PBS, samples were post-fixed in 2% glutaraldehyde in PBS for 20 min, washed and the ultra-small gold particles were silver-enhanced for 20 min at RT with AURION R-Gent Silver Enhancement for Electron Microscopy (EMS, Hatfield, PA, USA) following the manufacturer’s indications. Later, samples were post-fixed with 0.5% OsO_4_ in PBS for 20 min at 4°C and washed with PBS, dehydrated through a graded series of ethanol and embedded in Epoxy EMbed-812 resin (EMS, Hatfield, PA, USA). Ultrathin sections were obtained with an Ultracut E ultramicrotome (Reichert/Leica; Leica Microsystems, Wetzlar, Germany), contrasted with uranyl acetate and lead citrate and analyzed using a Tecnai Spirit Twin 120 kv electron microscope with a CCD Gatan Orius SC200D camera with DigitalMicrograph software. Electron microscopy was performed with the assistance of Electron Microscopy Service-NUCLEUS (University of Salamanca, Salamanca, Spain).

### 2.8. Extracellular Media Processing and Extracellular Vesicles Isolation

Primary microglia, isolated as described above and grown in 10 cm diameter Petri dishes (78.5 cm^2^), were washed with PBS at 37 °C. Cells were subjected to different treatments in RPMI medium without FBS for 24 h, as indicated above.

Extracellular media preparations: Conditioned media was centrifuged (200× *g*, 5 min, RT) to remove floating cells, and the supernatant was centrifuged (2820× *g*, 10 min, 4 °C) to pellet debris. Cell and debris-free conditioned media were then concentrated using Amicon Filters 10K (Merck-Millipore, Darmstadt, Germany) by centrifugation (2820× *g*, 60 min, 4 °C). Protein concentrations were BCA-quantified, and 80 μg of protein were analyzed by immunoblot.

For extracellular vesicles (EVs) isolation, conditioned media were collected and centrifuged (450× *g*, 10 min, 4 °C) to pellet cells, and the supernatant was subsequently filtered through a 0.2 μm PES filter to remove debris. Cell and debris-free conditioned media were ultracentrifuged (100 Ti rotor, 120,000× *g*, 75 min at 4 °C) to pellet EVs. The resulting pellets were resuspended in protein sample buffer and analyzed by immunoblot.

### 2.9. Statistical Analysis

Statistical analyses were performed with SigmaPlot v.11.0 software (Systat Software, Inc., San Jose, CA, USA). A *p*-value of <0.05 was set as a threshold for significant changes. The tests used for each experiment are stated in figure legends.

## 3. Results

### 3.1. Phylogeny of Metalloendopeptidases Belonging to Clan ME

**Figure 1:**
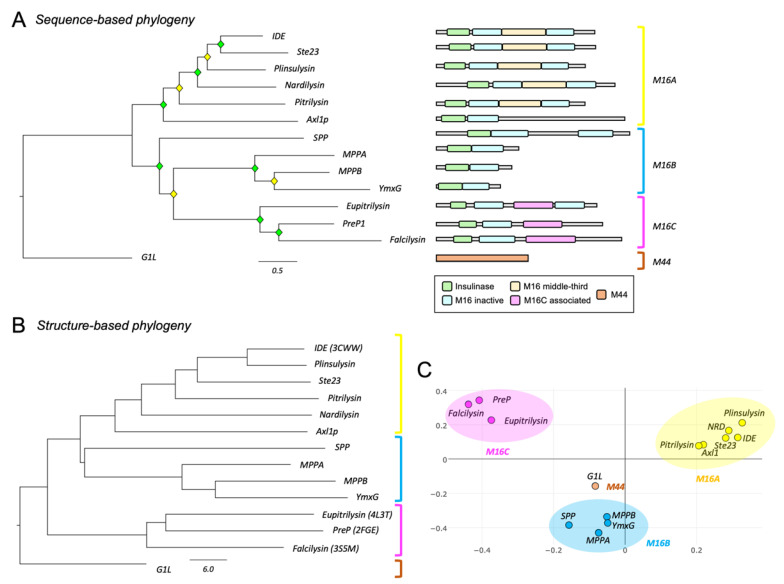
Phylogenetic analyses of the clan ME of metallopeptidases. (**A**) Phylogenetic relationships based on protein sequence alignments. A Maximum Likelihood (ML) phylogenetic tree was constructed with 14 representative metallopeptidases of clan ME, using a substitution model LG + F + I + G4, according to Bayesian Information Criterion (BIC). Diamonds label nodes with a bootstrap value >60% (yellow) and >80% (green). Scale branch length represents number of amino acid substitutions per site. Domain arrangement for each protein, retrieved from Pfam and HMMER databases, is shown on the right. (**B**) Phylogenetic relationships of clan ME proteins based on protein structure. Structures were retrieved from Protein Data Bank (PDB) or predicted using either AlphaFold or SwissProt 3D online servers. Dendrogram depicts the relationships of clan ME metallopeptidases structurally aligned. Scale branch represents distances obtained from the Dali similarity matrix. (**C**) Correspondence analysis clustering proteins with the most similar structural neighbor near each other.

Primary structure analyses of clan ME metalloendopeptidases clearly separate the selected proteins into their corresponding families (Figure 1A, left), revealing a good correlation between the functional classification proposed by Rawlings and Barrett [4] and their sequence-based relationships. The comparison of functional domains of each protein (Figure 1A, right) shows distinctive patterns among families. The M16B proteins present only one insulinase and one M16 inactive domain, except for SPP, which might have undergone a duplication event resulting in a protein twice the length of other M16B proteins. The M16C family includes also an M16C-associated domain, while the M16A family has a third domain in the middle. In all cases, catalytic activity resides at the N-terminus (insulinase domain). Contrary to amino acid sequence, the protein tertiary structure is highly conserved among M16 members (Supplementary Materials File S2—Figure S2). The protein structure-based dendrogram also correctly separates the different families (Figure 1B,C).

### 3.2. The M16A Family Is Highly Conserved across Evolution

Our search for IDE homologous proteins rendered results in all kingdoms, from *Archaea* to *Eukarya*. Since M16A are multidomain proteins, our initial searches produced many hits that had to be manually curated: many candidates were metalloproteinases but had only one or two domains. Following our criteria (see Methods section), we created a M16A family database including 2165 sequences. The presence of IDE-related proteins in all kingdoms is remarkable, with a unique IDE homolog in *Archaea* and many IDE-like proteins in *Proteobacteria*, while other prokaryotic phyla do not present similar proteins. Surprisingly, M16A proteins are also found in various *Mimiviruses*. In *Eukarya*, IDE-related proteins are widely represented.

**Figure 2:**
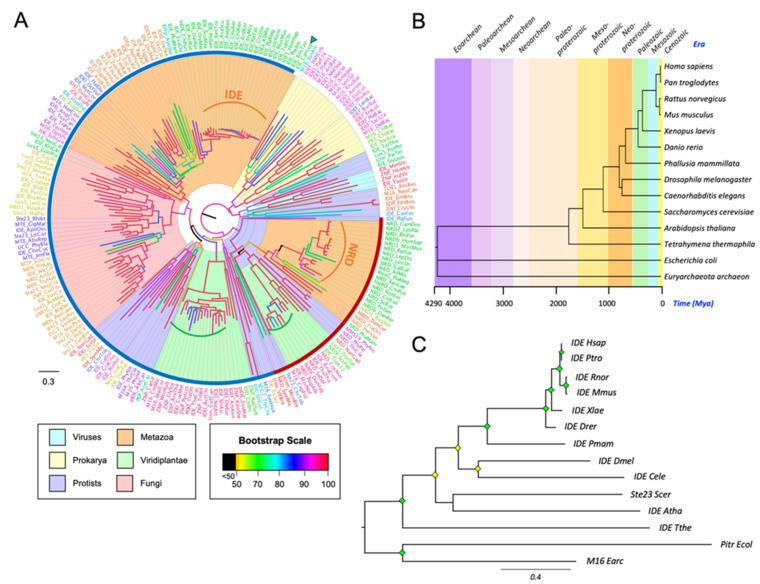
Global phylogeny of M16A proteins. (**A**) A phylogenetic tree was reconstructed from a protein sequence-based MSA of 216 IDE homologs. The substitution model LG + I + G4 was chosen according to BIC, and the tree was inferred using an ML method. Major species groups are highlighted by different colors as indicated. Arrowhead points to the only *Archaea* representative. Brown and blue arches encompass NRD and IDE monophyletic clades, respectively. Curved brackets highlight clades with short branches (low divergence) for some M16A plant proteins (green brackets) and for some members of the IDE and NRD clades (orange brackets). (**B**) Phylogeny and evolution timescale of the 14 organisms selected for IDE phylogeny. (**C**) Phylogenetic tree of 14 IDE-like proteins reconstructed from a protein sequence-based Multiple Sequence Alignment (MSA) and an ML method (substitution model LG + I + G4, chosen according to BIC). Diamonds label nodes with a bootstrap value >60% (yellow) and >80% (green). Scale branch length represents number of amino acid substitutions per site. Members of the prokaryote kingdom were selected as the outgroup.

From our M16A database, we selected 216 sequences to infer a global tree (Figure 2A). All prokaryotic sequences cluster together, with the *Archaea* sequence (arrowhead) separated from bacterial proteins. These taxa present long branches, suggesting that M16A proteins have undergone functional explorations leading to strong sequence divergence in these organisms. The viral sequences are clustered within protists, which makes perfect sense since *Mimiviruses* are protist viruses that have large genomes with many horizontally acquired genes. The protist sequences are split into various groups, which is reasonable since protists are a diverse collection of unicellular organisms with puzzling affinities. The *Viridiplantae* (plants and green algae) sequences appear in two different groups, and a notable shortening of some of these branches (green brackets in Figure 2A) suggests that the function of the protein is being fixed by selection constraints. The fungal sequences are grouped all together and separated by phyla. Interestingly, in *Metazoans,* IDE and its homologous protein NRD are clearly separated into two clusters that show very short branches (orange brackets in Figure 2A) in *Chordata*. Based on its tree position, NRD appears more related to bacterial M16A proteins, while IDE stems as a separate family member with the advent of protists.

To deepen into IDE evolution, 14 representative organisms were selected. The phylogenetic relationship and evolutionary timescale of the selected organisms was estimated with TimeTree [42] (Figure 2B), and the protein sequence-based phylogeny for IDE is shown in Figure 2C. The high conservation in vertebrate species, compared with other chordates and other metazoan phyla, is indicative of selective constraints acting on IDE function early in vertebrate evolution.

### 3.3. IDE and NRD Are Paralogous Proteins with Different Gene Structure

**Figure 3:**
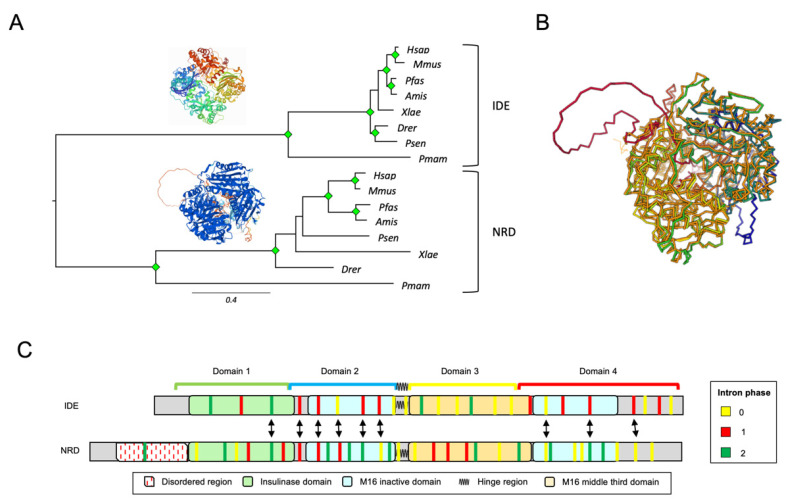
IDE and NRD comparison in chordates. (**A**) Phylogenetic relationships of IDE and NRD in 8 selected species of *Chordata*. The tree was built from protein sequence-based MSAs and an ML method (substitution model: LG + G4). Protein structures depicted are 3CWW for IDE and a prediction from the AlphaFold database for NRD. Green diamonds label nodes with a bootstrap value >80%. (**B**) Superimposition of 3D structures of IDE (in orange) and NRD (rainbow colors) resultant from a structural alignment on the Dali Server. (**C**) Intronic architecture of *IDE* and *NRD* genes mapped onto a multiple protein sequence alignment in which proteins are represented by their constituent domains. Equivalent introns (with a distance less than 5 residues) are pointed out by arrows.

We focused on the comparison of IDE and its homologous protein NRD in *Chordata*, combining information coming from protein sequence alignments, exon–intron structure and protein structure comparison. We selected eight chordate species expressing both IDE and NRD and whose genomes were sequenced with good coverage. Initially, we performed an MSA with the selected protein sequences and observed that IDE proteins possess shorter branches than NRD, indicating that IDE function is subject to higher selective pressures (Figure 3A). Then, we used the Dali server to structurally align IDE and NRD (3D superimposition shown in Figure 3B) and found that both proteins have an almost identical 3D structure, with the only exception of two loops in NRD, corresponding to disordered regions rich in acidic residues at the N-terminus (Figure 3B,C). Finally, we compared IDE and NRD at the gene level, analyzing the exon–intron structure of 26 sequences. The full MSA of the selected sequences, as well as their intronic architecture, can be found in Supplementary Materials File S2—Figure S3. When comparing the intronic architecture of human *IDE* (25 exons, chromosome 10) and human *NRD* (33 exons, chromosome 1), we observed that several intron positions align well when mapped onto the MSA (double arrows, Figure 3C), which supports the paralogy of these proteins and their origin by gene duplication. The species representation of IDE and NRD, with NRD located close to prokaryotes in the general M16A tree, strongly supports the origin of *IDE* as a relatively recent duplicate, maintaining some of the ancestral gene structure.

### 3.4. M16A Proteins Topogenesis Changed during Evolution

**Figure 4:**
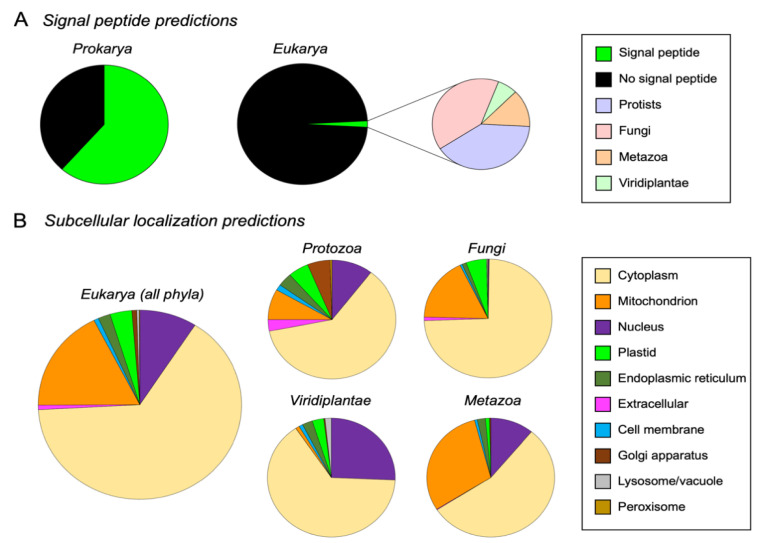
Bioinformatic predictions on M16A cellular localization. (**A**) N-terminal signal peptide predictions in prokaryotic and eukaryotic M16A sequences. A total of 61.5% of IDE-like sequences have signal peptide in prokaryotes, while only 1.7% of eukaryotic sequences possess this protein trait. (**B**) Subcellular localization predictions for IDE-like eukaryotic proteins, depicted collectively in all phyla (left graph) or individually in each phylum (right graphs).

An in silico analysis of M16A proteins using SignalP5.0 revealed that a high percentage (61.5%) had N-terminal signal peptides in *Prokarya.* It is worth noting the great variability found inside *Gammaproteobacteria*, where the signal peptide was totally absent in some orders (*Aeromonadales*), while it was a predominant feature in others (*Alteromonadales*) (Figure 4A and Supplementary Materials File S3—Tables S5 and S6). On the contrary, the number of M16A eukaryotic proteins predicted to have a signal peptide were surprisingly low (1.7%) (Figure 4A and Supplementary Materials File S3—Tables S5 and S6) and was absent in the 308 sequences studied from *Chordata*. Taken together, these results indicate a shift during evolution in the mechanism for exporting M16A proteins to the extracellular space, with some prokaryotic sequences presenting N-terminal signal peptides required for canonical secretion, while the trend is inverted in eukaryotes. A parsimonious explanation for this pattern is that some M16A proteins of prokaryotes and unicellular eukaryotes bear topogenic sequences that facilitate their transport across membranes to the periplasm, extracellular space or to cell organelles, while multicellular organisms have lost those sequences. Among the few eukaryotic sequences that possess signal peptides, 80% belong to protists and fungi, with very few proteins with signal peptide in plants and arthropods. This opens the possibility that some M16A sequences with signal peptides found in multicellular organisms might be of prokaryotic origin due to endosymbiosis, horizontal transfer or even to microbiota contamination.

The subcellular localization of eukaryotic M16A proteins (IDE and NRD clades) from our database was predicted using DeepLoc1.0. From a total of 1815 eukaryotic proteins analyzed, the majority (65%) were predicted to be cytosolic. Other subcellular locations found were the mitochondrion (17.6%), nucleus (9.1%), plastids (3.5%), endoplasmic reticulum (1.9%), Golgi apparatus (0.8%), extracellular (0.8%), lysosome (0.4%) and peroxisome (0.1%) (Figure 4B, left graph, and Supplementary Materials File S3—Table S7). In accordance with this, 97.7% of these proteins were predicted to be soluble, while only 2.3% were predicted to be membrane-associated (Supplementary Materials File S3—Table S7). Analyzing the predicted subcellular localizations individually for each kingdom, the cytoplasm was again the preferred location (Figure 4B, right graphs). However, interesting differences between groups could be observed. Protists exhibited the greatest diversity of localizations, which agrees with the proposed functional explorations in this group appreciated in the global phylogeny (Figure 2A). In protists, the cytoplasmic localization was followed by the nucleus and mitochondrion, but the most striking predictions were the extracellular and cell membrane locations, hardly seen in other groups, which might be indicative of a bona fide exportation pathway. Fungi showed a dramatic decrease in the number of predicted subcellular localizations, with clear predominance of the cytoplasm, followed by mitochondrion and plastids. The *Viridiplantae* kingdom presented different results, with many proteins predicted to be cytosolic, with the nuclear location as second in frequency. Finally, metazoan proteins were preferentially located in the cytoplasm, followed by mitochondria and the nucleus as alternative locations (Figure 4B). These results strongly support the hypothesis that IDE-like proteins are mainly cytosolic.

### 3.5. Microglial Cells Display IDE in Cytosol, Membranes and Lipid Rafts

After in silico studies, we analyzed IDE subcellular localization and traffic. We decided to focus on microglia since they are cells that have many physiological states and can switch from a surveillance (“resting”) state, with high motility and membrane reorganization, to a secretory (“activated”) state in response to harmful stimuli in the nervous system.

**Figure 5:**
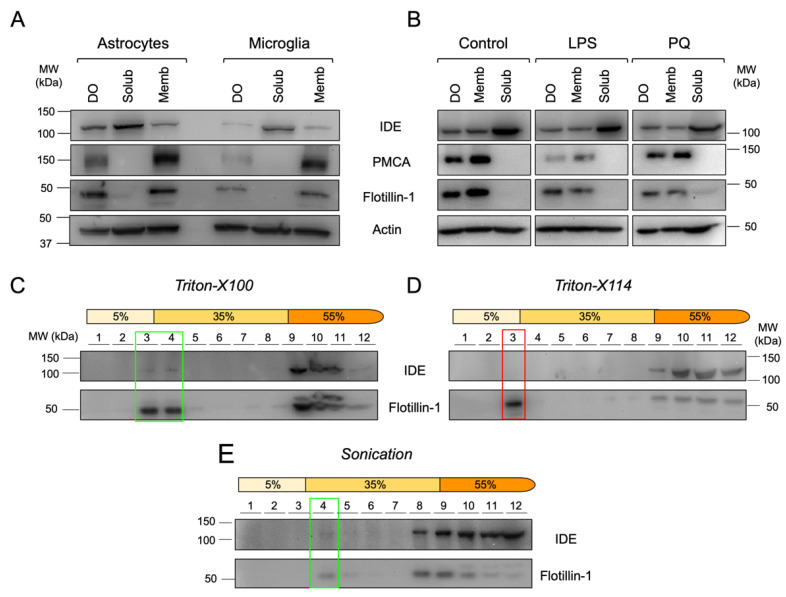
IDE is stably associated to membranes of primary glial cells and to membrane microdomains with specific physicochemical properties in the microglial cell line BV-2. (**A**) Immunoblot analyses of primary glial cells upon centrifugal fractionation in dense organelles (DO), membrane (Memb) and soluble (Solub) fractions. (**B**) Immunoblot analyses of BV-2 microglial cells treated with different stimuli (100 ng/mL LPS or 25 μM PQ for 24 h) and fractionated in DO, Memb and Solub fractions. (**C**–**E**) Immunoblot analyses of membrane fractionation into lipid rafts and non-raft domains using different methods: Triton-X100 (**C**), Triton-X114 (**D**) and sonication (**E**). Flot-1 was used as a lipid raft marker. Rectangles highlight the lipid raft fractions.

We performed biochemical cell fractionation of primary glial cultures enriched in microglia or astrocytes and analyzed the resultant fractions by immunoblot. In both microglia and astrocytes, IDE was mostly cytosolic as our bioinformatics analysis predicted, but a small fraction of the protein was associated with membranes and dense organelles (including nuclei, endoplasmic reticulum and mitochondria) (Figure 5A). These results indicate that IDE association with plasma membrane is independent on the glial cell type, although the amounts of membrane-bound IDE can slightly vary between cell types.

With the antecedents stated above, a relevant question is whether IDE association to membranes is stable upon exposure of cells to inflammatory (LPS) or oxidative stress (PQ) conditions. In microglial BV-2 cells, IDE was also mostly cytosolic, but a significant fraction appeared associated with membranes and, to a lesser extent, with dense organelles. This IDE partition between soluble and membrane fractions was independent of the stimulus (Figure 5B) and confirms the ubiquitous subcellular distribution of IDE in microglial cells and its stable association with membranes.

Lipid rafts are specific microdomains in cellular membranes, enriched in cholesterol and sphingolipids, and involved in many cell-signaling processes. To test whether IDE-membrane association occurs in particular membrane microdomains, we isolated lipid rafts by treating BV-2 membrane preparations with different chemical (Triton-X100 and Triton-X114) and mechanical (sonication) methods, using Flotillin-1 as a lipid raft marker (Figure 5C–E). IDE was detected in Triton-X100 and sonication-resistant membrane domains (Figure 5C,E, respectively), while it was absent in Triton-X114-resistant membrane domains (Figure 5D). In all cases, IDE protein was also detected in non-raft fractions. These results demonstrate that a small proportion of IDE is present in particular types of membrane microdomains, indicating that IDE association to lipid rafts is not universal but specific to the physicochemical properties of membranes. Additionally, our results support a scenario where IDE can switch from non-raft to raft membrane domains.

### 3.6. IDE Associates to Membranes by Their Cytosolic Side

Having demonstrated that IDE associates with the plasma membrane, the obvious next step to understand its functions is to establish on which side of the membrane this interaction occurs. To address this point, we performed immunocytochemistry and electron microscopy experiments.

**Figure 6:**
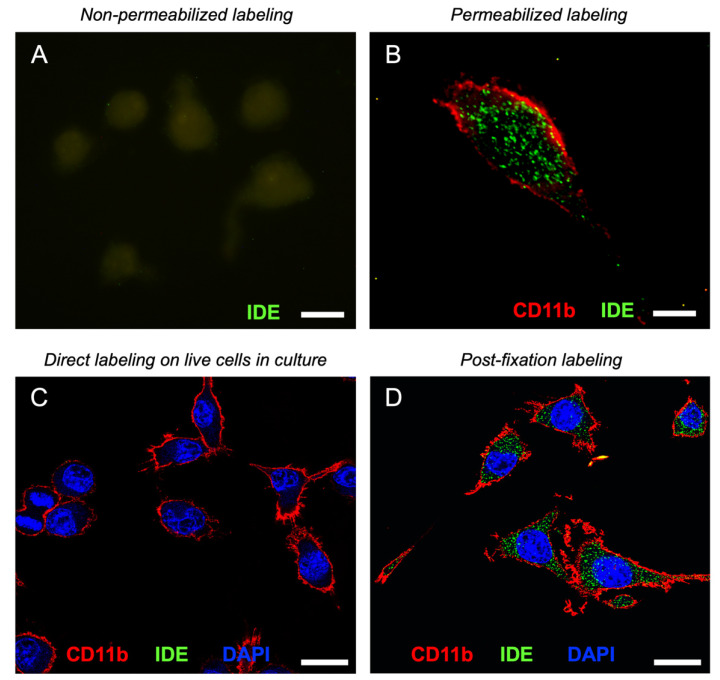
IDE associates to membranes only at the cytoplasmic side. (**A**,**B**) Representative fluorescence microscopy images of IDE signal in non-permeabilized (**A**) and permeabilized (**B**) BV-2 cells. Only background autofluorescence can be detected in (**A**). Image (**B**) shows a deconvolved projection of a Z-stack. (**C**,**D**) Representative confocal sections of IDE and CD11b signal after direct labeling of live cells in culture (**C**) and in post-fixation and permeabilization labeled cells (**D**). Calibration bars in (**A**,**B**): 10 µm, (**C**,**D**): 20 µm.

First, we explored different settings on immunocytochemistry experiments to try to decipher IDE subcellular localization in BV-2 microglial cells. Non-permeabilized conditions did not present IDE labeling (Figure 6A), demonstrating that IDE was not present at the extracellular side of the plasma membrane. On the other hand, under permeabilizing conditions, IDE signal was present inside the cells in a punctate pattern (Figure 6B), suggesting that IDE is cytosolic and might be associated with vesicular organelles.

To further confirm the absence of IDE on the cell surface, we performed direct immunolabeling experiments on live cells in culture using CD11b, a microglial surface integrin, as a positive control. Confocal images showed no IDE signal, while CD11b yielded a robust labeling all over the cell surface (Figure 6C). However, after fixation and permeabilization steps, abundant IDE signal was evident inside the cells in a clear punctate pattern (Figure 6D). These results indicate that microglial IDE is associated with membranes exclusively at the cytoplasmic side, with no detectable IDE exposed on the cell surface. Supplementary Materials File S2—Figure S4 shows the validation of IDE antibody using WT and IDE-KO microglia, immunocytochemistry negative controls and more examples of IDE labeling in microglial cells.

### 3.7. IDE Is Found in Multivesicular Bodies and Their Microvesicles in Secretory Microglia

**Figure 7:**
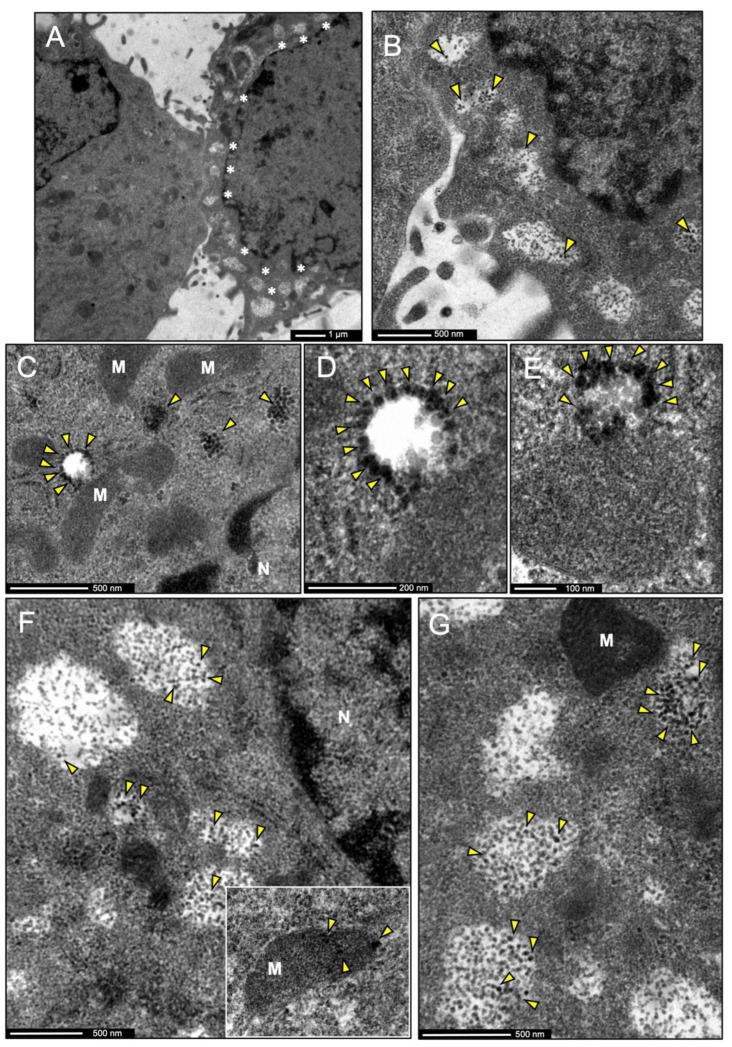
Immunoelectron microscopy micrographs of the subcellular localization of IDE in BV-2 microglial cells. IDE labeling is shown by means of silver-enhanced gold particles. (**A**) Representative image of the two types of BV-2 cells found in EM sections, some with scarce vesicles (left) and others with numerous vesicles (right, pointed out by white asterisks). IDE labeling was only detected in cells with multiple vesicles. (**B**) Inset from (**A**), showing a closer view of the vesicles and the IDE signal (arrowheads). (**C**–**E**) Representative images of immunogold labeling of IDE in small multivesicular bodies (MVBs) and their nascent and internal microvesicles (arrowheads). (**F**,**G**) Images showing larger MVBs, generally found closer to the cell surface (as in **B**), with IDE labeling in microvesicles inside them (arrowheads). The inset in F shows the only example of mitochondrion with IDE signal found in our samples. Abbreviations: M = Mitochondria, N = Nucleus. IDE labeling is pointed by yellow arrowheads.

The subcellular localization of IDE in microglial cells was further confirmed by immunoelectron microscopy. Two different physiological states were clearly observed in BV-2 cultured cells. Some of them presented very few vesicles and were probably in their surveillance state. Other cells, presenting numerous vesicles in the vicinity of the plasma membrane, could be in an “activated” secretory state (Figure 7A). IDE labeling was found in cells with multiple vesicles, suggesting a role for IDE when the microglial cells acquire a phenotype within the spectrum of active secretory responses. IDE was mostly cytosolic, but was also found in large vesicles clustered in close proximity to the plasma membrane (Figure 7B). IDE-positive vesicles have the characteristics of multivesicular bodies (MVBs), morphologically distinctive by a mean diameter of 200–500 nm and characterized by an electron lucent matrix and the presence of microvesicles formed by invagination on its surface. IDE was found on the external side of MVBs or in their intraluminal vesicles (Figure 7C–E). Large MVBs with IDE-positive microvesicles were found close to the cell surface, probably on their way to secrete their content (Figure 7F,G). After exhaustive exploration, IDE was not found inside any other organelle, except for one occasional signal in mitochondria (inset in Figure 7F).

These results reinforce our in silico predictions about IDE subcellular localization and the results obtained by immunofluorescence microscopy being mostly cytosolic in microglial cells. The scarce mitochondrial location of IDE predicts that microglia do not significantly express a longer IDE isoform (IDE-Met^1^), described to contain a mitochondrial targeting sequence [12]. Furthermore, the striking feature of the preferential location of IDE in MVBs and their microvesicles suggests a non-canonical extracellular vesicle mediated secretion of IDE to the extracellular space. These results led us to analyze IDE exportation under different stimuli relevant for microglial responses.

### 3.8. IDE Exportation in Extracellular Vesicles Is Regulated by the Activation State of Microglial Cells

**Figure 8:**
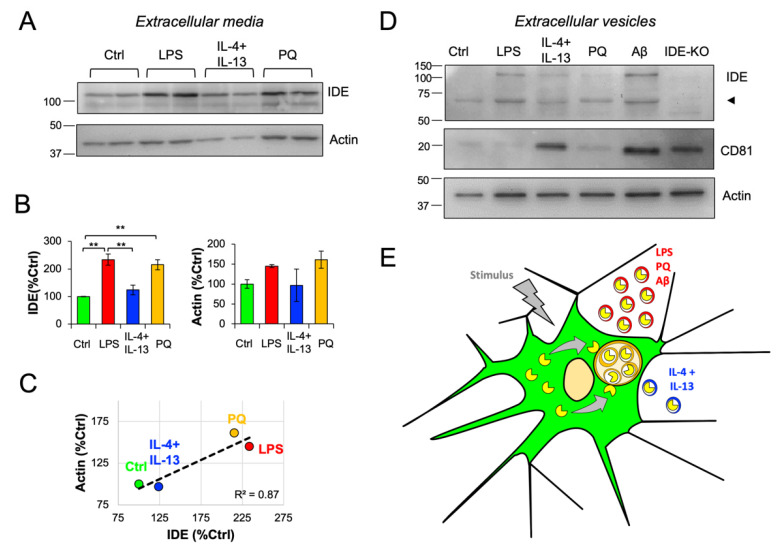
IDE exportation in extracellular vesicles is dependent on microglial activation state. Primary microglia were treated for 24 h with different stimuli, and conditioned media were collected and analyzed. (**A**) Immunoblot analyses of concentrated conditioned media. (**B**) Quantification of extracellular IDE and actin proteins from n = 3 independent experiments. Statistical differences were assessed by one-way ANOVA (IDE: *p* < 0.001 Actin: *p* = 0.25) followed by post-hoc Bonferroni *t*-tests. **, *p* < 0.01. (**C**) Scatterplot representing the amount of extracellular IDE and actin. Both proteins show a positive correlation (linear regression fit: y = 0.45x + 49.28; R^2^ = 0.87). (**D**) Immunoblot analyses of extracellular vesicles (EVs) preparations. Two pools of conditioned media originating from 4 independent cultures were analyzed. EVs from IDE-KO microglia were included as negative controls for the IDE antibody. The arrowhead points out a putative “half-IDE” form of 60 kDa. CD81 and actin were analyzed as EVs markers. (**E**) Proposed mechanism for IDE exportation in microglial cells. Under unstimulated conditions, IDE is mostly cytosolic. When stimulated, microglia changes into a “secretory active” state, produces numerous MVBs and IDE associates with their membrane and becomes internalized in microvesicles. This allows IDE to be exported outside the cells in small extracellular vesicles (exosomes). Such IDE exportation depends on stimuli relevant in physiological conditions and upon aging and neurodegeneration.

We tested whether stimuli relevant for microglial cells, able to regulate their physiological state, could be implicated in regulating the export of IDE to the extracellular space. Based on previous reports describing IDE in exosomes [17,18,25], we initially explored the presence of IDE in concentrated extracellular media from primary microglia exposed to different stimuli for 24 h. We found that IDE exportation from microglia was regulated by the activation state of the cells, increased under LPS and PQ stimuli, and reduced by IL-4 + IL-13 treatment (Figure 8A,B). Since actin has been reported in extracellular vesicles (EVs) [43], we quantified the extracellular actin (Figure 8A,B) to estimate the secretory activity of microglia, which resulted higher upon LPS and PQ stimulations. Furthermore, extracellular IDE and actin showed a strong positive correlation, clearly related with the microglial activation state (Figure 8C). Such correlation supports that IDE is not directly secreted to the media but must be present in EVs, containing an actin cytoskeleton associated to their membrane. These results suggest that microglial IDE is exported in actin-positive EVs, and the amount of IDE present in these EVs is regulated by the activation state of microglia.

To further investigate this hypothesis, we purified EVs from conditioned media from primary microglia treated with different pro-/anti-inflammatory stimuli, this time including oligomeric Aβ as a relevant stimulus and EVs from IDE-KO microglia as negative controls. We confirmed the presence of IDE in microglial EVs-enriched fractions (Figure 8D). Strikingly, we found that besides the expected 110-kDa IDE band, there was another band around 60 kDa, whose specificity was validated by its absence in the IDE-KO sample. The origin of this “half-IDE” isoform might be explained by the breakdown of the protein by its hinge region, which would produce two 55–60 kDa peptides. Interestingly, full-length IDE only seemed to be present after stimulation, while the shorter isoform was present in all conditions. Further research is needed to contrast this hypothesis. To further characterize our EV preparations, CD81 and actin were included in the immunoblot analysis. In contrast to actin, which showed a homogeneous presence between conditions, CD81 exhibited great differences between samples (Figure 8D). Remarkably, CD81 was reported to be upregulated in microglial cells after a central nervous system injury [44], which suggests that this protein might be differentially regulated by the microglial activation state. Under control conditions, very little CD81 was detected in EVs, while upon both IL-4 + IL-13 and Aβ, microglial EVs were qualitatively enriched in CD81, which suggest the upregulation of this tetraspanin when microglia are polarized to a “phagocytic-like” activation state, as opposed to EVs produced by LPS- and PQ-stimulated microglia (Figure 8D). Furthermore, such differential expression pattern might be indicative of the production of different types of vesicles upon different stimuli-induced microglial activation states.

These results reinforce the idea that IDE is secreted in MVB-derived EVs but also demonstrate for the first time that this mechanism of IDE exportation is modulated by different polarization states of the microglial cells and by stimuli that are particularly relevant to neurodegenerative conditions in the brain (Figure 8E).

## 4. Discussion

In this work, we first addressed IDE subcellular localization and exportation mechanisms from an evolutionary perspective by analyzing the molecular evolution of the M16A family. We found that M16A metalloendopeptidases are highly conserved, being present throughout the tree of life from archaea and bacteria to metazoans. Two main paralogous protein clades are apparent in metazoans, IDE and NRD, whose divergence can be traced in the global tree to ancient M16A gene duplications in protists. According to the tree topology, the NRD clade shares more sequence similarity to the ancestral protist and prokaryotic M16A proteins. The IDE clade appears diversified in multicellular organisms, though preserves strong sequence similarity in chordates, possibly due to newly acquired biological functions. 

Our in silico studies on M16A proteins revealed that N-terminal signal peptides are predicted for a group of extant prokaryotes and unicellular eukaryotes, while this targeting motif is mostly absent in multicellular organisms. This finding strongly supports that this trait got lost during the evolution of IDE ancestors, which tallies with the fact that signal peptides become lost more often in the course of evolution than they are gained, with these events preferentially occurring in the transition from free-living bacteria to endosymbionts [45]. The cell location of clan ME metalloproteases further supports this hypothesis: bacterial pitrilysin is periplasmic [46], eupitrilysin and MPPs are found in mitochondria [47,48], SPP in the stroma of chloroplasts [49] and PreP1 in both mitochondria and chloroplasts [50]. Although eukaryotic M16A proteins lack signal peptides, we must consider other “non-canonical” secretory mechanisms that could underlie extracellular functions for these proteases.

Focusing on the subcellular localization of IDE in microglia, we found that it partitions between soluble and membrane fractions, as previously described in neurons [22]. However, in contrast to some studies that detected IDE on the cell surface of lymphocytes and hepatocytes [23], pancreatic acini cells [24] and neurons [22], our biochemical and immunocytochemical analysis in microglial cells found IDE association at the cytoplasmic side of cellular membranes, with no IDE exposed on the cell surface. These results agree with our bioinformatic analyses, as a lack of signal peptide argues against IDE association to the extracellular side of the plasma membrane. Interestingly, we compared different methods for lipid raft isolation and found that a small fraction of membrane-associated IDE is found in Triton-X100 and sonication-resistant microdomains, while it is absent on Triton-X114 resistant domains. These results suggest a transient association of IDE with microglial lipid rafts, which is in agreement with the faster IDE turnover described for membrane-associated IDE in comparison with the cytosolic pool [22], and supports a dynamic association of IDE to membrane microdomains.

Some studies have reported the secretion of IDE to the extracellular space by microglia [8,51], neurons [18,21,52] and astrocytes [53]. However, it has been recently called into question the secretion of IDE, arguing that IDE in conditioned media could result from loss of cell integrity rather than specific protein secretion [54]. We have used two strategies to rule out the detection of IDE from dying cells: (1) we have analyzed cell-debris free conditioned media (by a series of centrifugations at the collection step) and (2) we isolated EVs from primary microglial cultures. In both setups, we actually detected extracellular IDE. Furthermore, a 60 kDa “isoform” of IDE was detected in EV preparations. The antibody used in this work (rabbit anti-IDE, #AB9210, Merck-Millipore, Darmstadt, Germany) is a polyclonal antibody whose epitope is located at amino acids 150–300 of a rat IDE sequence; therefore, the putative “half-IDE” that we detected corresponds to the N-terminal portion of IDE. In vitro experiments using limited proteolysis described that trypsin digestion of human IDE results in two proteolytic fragments, corresponding to a 57.5 kDa IDE-N (domains 1–2) and a 55.8 kDa IDE-C (domains 3–4) [55], and our results constitute, up to our knowledge, the first detection of such IDE proteolysis in cultured cells. A relevant question is whether such IDE-N “isoform”, which contains the insulinase domain and the catalytic site, has catalytic activity or not. In this regard, Li and colleagues reported that IDE-N only has about 2% of the activity of full-length IDE, but the mixing of IDE-N with IDE-C in vitro resulted in the reassembly of both parts to make a full IDE protein [55]. These data support that EV-associated shortened IDE, which is found constitutively in EVs, could be functional after reassembly with the IDE-C half. More studies are needed to further assess the biological meaning and potential functionality of this EV-associated IDE-N “isoform”. In any case, our results show that extracellular IDE is accounted for by EVs secretion and demonstrate for the first time that the amount of IDE present in EVs is regulated by the activation state of microglial cells, with more IDE being released under LPS, PQ and Aβ oligomer treatments, while IDE exportation was decreased under IL-4 + IL-13 treatment.

Regarding Aβ, our results agree with a recent paper describing that microglia partially clears soluble Aβ peptides from the extracellular space by secreting IDE [51]. In our electron microscopy screening, we described a prominent IDE presence in MVBs of highly secretory cells, which is in agreement with the increased IDE secretion upon microglial activation with different treatments. Our results confirmed that IDE is exported in EVs, which are probably MVB-derived exosomes. However, the differences in CD81 content of IDE-positive EVs purified from primary microglia opens the possibility of a secretion by ectosomes (derived from plasma membrane) regulated by relevant stimuli. Given the in vitro Aβ-degrading ability of IDE, one question that remains to be solved is how IDE interacts with Aβ since IDE would necessarily be inside EVs while extracellular Aβ would be outside these vesicles. A plausible hypothesis is that IDE might be released upon breakage of exosomes, an event that has been described for certain EVs and involves the discharge of their contents into the extracellular space [56]. A different scenario has been proposed for astrocytes to extracellularly release IDE via an unconventional autophagy-mediated secretion [53], although it was not demonstrated how a double bilayer vesicle fuses with the plasma membrane to release its protein content. Yet an alternative mechanism could imply an endocytosis of Aβ peptides or oligomers by microglia followed by targeting to MVBs, where IDE and Aβ would be in separate compartments. A subsequent fusion with lysosomes could favor the encounter of IDE and Aβ after intraluminal vesicle membrane degradation. However, the acidic pH of this lysosomal compartment could both inhibit Aβ binding to IDE [9] and jeopardize IDE proteolytic activity [57]. Mounting evidence proposes that IDE function goes far beyond its proteolytic role as a regulator of insulin and Aβ levels, and, in fact, IDE has recently been proposed as a moonlight enzyme that plays similar roles to heat shock proteins and chaperons and even modulates the ubiquitin proteasomal system [58].

In summary, this work provides a detailed investigation of IDE cellular biology, from its origin and molecular evolution to its subcellular localization in microglia. Our results demonstrate that IDE secretion is mediated by EVs originating from MVBs and that such an exportation mechanism is modulated by the polarization phenotype of microglial cells and by stimuli that are particularly relevant to neurodegenerative conditions in the brain. IDE location in the different activation states of microglia becomes, therefore, a relevant issue to focus future research if we are to understand its physiological function or to control its therapeutic potential.

## Figures and Tables

**Table 1:** Proteins for clan ME analysis.

Name	Family	Organism	Sequence ID
Pitrilysin	M16A	*Escherichia coli*	QIF70255
Nardilysin	M16A	*Homo sapiens*	O43847
IDE	M16A	*Homo sapiens*	P14735
Ste23	M16A	*Saccharomyces cerevisiae*	EWG94525
Axl1p	M16A	*Saccharomyces cerevisiae*	NP_015447
Plinsulysin	M16A	*Solanum lycopersicum*	NP_001233926
SPP	M16B	*Arabidopsis thaliana*	Q9FIH8
MPPA	M16B	*Homo sapiens*	Q10713
MPPB	M16B	*Homo sapiens*	O75439
YmxG	M16B	*Rickettsia prowazekii*	O05945
PreP1	M16C	*Arabidopsis thaliana*	Q9LJL3
Eupitrilysin	M16C	*Homo sapiens*	Q5JRX3
Falcilysin	M16C	*Plasmodium falciparum*	KAF4326450
G1L	M44	*Vaccinia virus*	P68493.1

## Data Availability

All data generated and analyzed during this study are included in this published article and its supplementary figure and table files.

## Supplementary Materials

The following are available online at https://www.mdpi.com/article/10.3390/cells11020227/s1, Supplementary File S1: File S1: IDE position-specific scoring matrix. Supplementary File S2: Figure S1: Genotyping of IDE-KO mouse colony; Figure S2: Structural alignments of clan ME metalloendopeptidases; Figure S3: Gene architecture comparison between 26 IDE and NRD proteins; Figure S4: Controls for microscopy experiments; Molecular phylogeny data S2: Trees output (text format). Supplementary File S3: Table S1: Sequences used in Figure 1; Table S2: Sequences used in Figure 2A; Table S3: Sequences used in Figure 2C; Table S4: Sequences used in Figure 3A; Table S5: SignalP predictions results; Table S6: SignalP summary per Phylum; Table S7: DeepLoc results.
