# Supplementary material for: Evolutionary Origin of Insulin-Degrading Enzyme and Its Subcellular Localization and Secretion Mechanism: A Study in Microglial Cells
Source: Cells. 2022 Jan 11;11(2):227. doi: 10.3390/cells11020227 (PMC8774118; doi:10.3390/cells11020227)
Supplement: Supplementary file 1 [file cells-11-00227-s001.zip › cells-1531819-suppl/Supporting Information File S2.pdf]

Supplementary Materials

Evolutionary Origin of Insulin-Degrading Enzyme and Its Subcellular Localization and Secretion Mechanism: A Study in Microglial Cells

Miriam Corraliza-Gómez [1,*], Concepción Lillo [2,3], Irene Cózar-Castellano [1,4], Eduardo Arranz [1], Diego Sanchez [1] and Maria D. Ganfornina [1]

1 Excellence Unit, Instituto de Biología y Genética Molecular, University of Valladolid-CSIC, 47003 Valladolid, Spain; irene.cozar@uva.es (I.C.-C.); earranz@uva.es (E.A.); lazarill@ibgm.uva.es (D.S.); opabinia@ibgm.uva.es (M.D.G.)
2 Instituto de Neurociencias de Castilla y León (INCYL), University of Salamanca, 37007 Salamanca, Spain; conlillo@usal.es
3 Hospital Virgen de la Vega-Instituto de Investigación Biomédica de Salamanca (IBSAL), 37007 Salamanca, Spain
4 Centro de Investigación Biomédica en Red de Diabetes y Enfermedades Metabólicas Asociadas (CIBERDEM), 28029 Madrid, Spain
* Correspondence: corraliza@ibgm.uva.es; Tel.: +34-983-184-814

TABLE OF CONTENTS

In .asn file (Supplementary File 1):

**File S1:** IDE position-specific scoring matrix.

In this file (Supplementary File 2):

**Figure S1.** Genotyping of IDE-KO mouse colony.
**Figure S2.** Structural alignments of clan ME metalloendopeptidases.
**Figure S3.** Gene architecture comparison between 26 IDE and NRD proteins.
**Figure S4.** Controls for microscopy experiments.
**Molecular phylogeny data S2:** Trees output (text format).

In Excel file (Supplementary File 3):

**Table S1:** Sequences used in Figure 1.
**Table S2:** Sequences used in Figure 2A.
**Table S3:** Sequences used in Figure 2C.
**Table S4:** Sequences used in Figure 3A.
**Table S5:** SignalP predictions results.
**Table S6:** SignalP summary per Phylum.
**Table S7:** DeepLoc results.

A

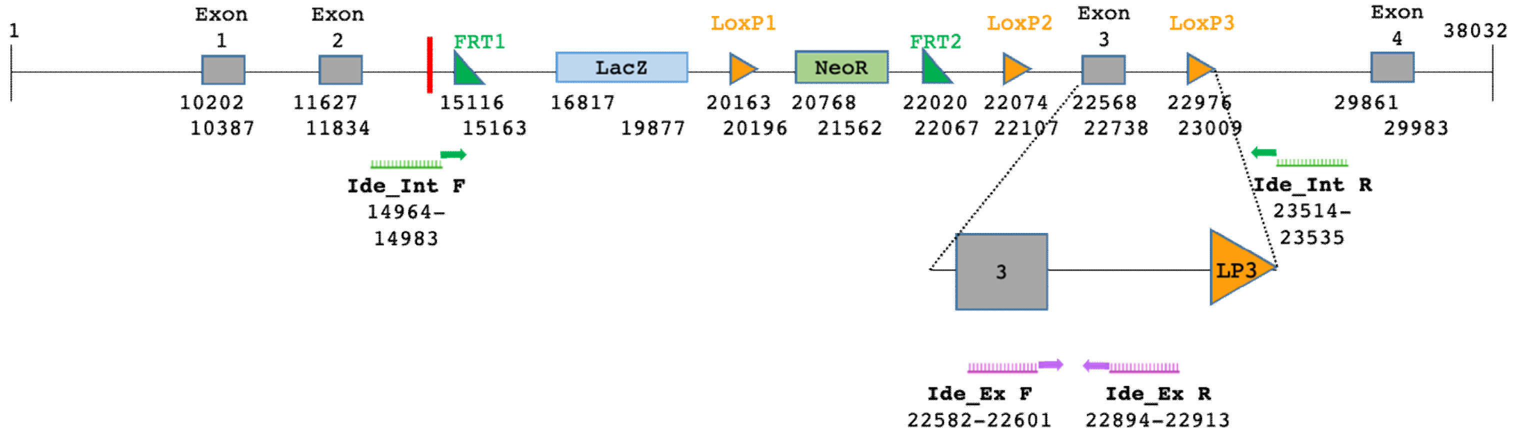

B

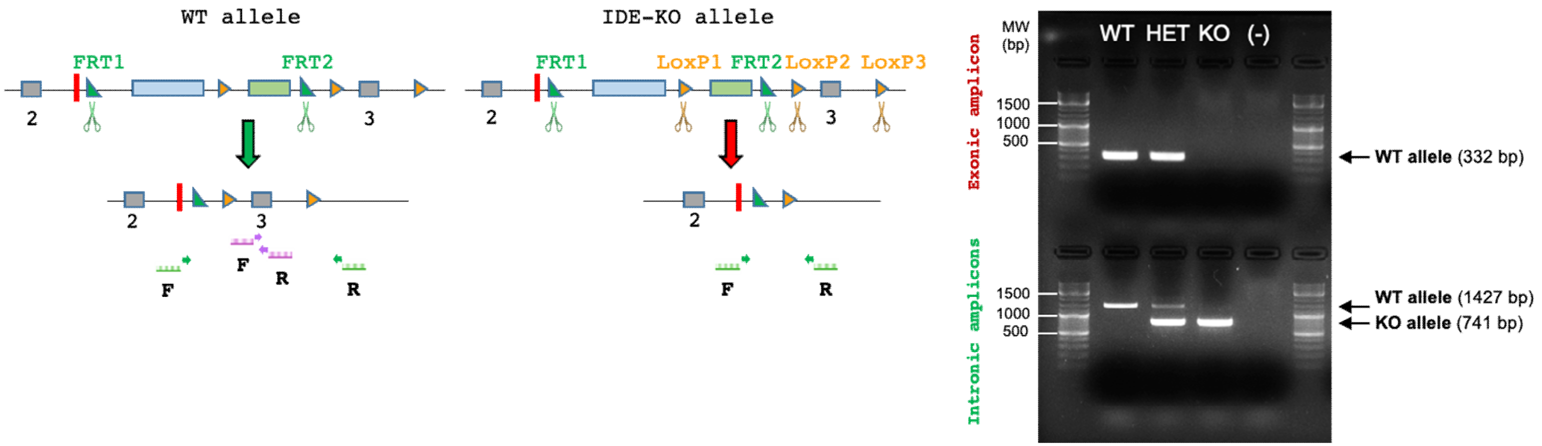

Figure S1. Genotyping of IDE-KO mouse colony. A) Scheme of the genomic map of mutant allele Ide-KO construct (NCBI GenBank JN963136.1) and the genomic localization of the primers designed to genotype IDE-KO mouse colony. **B)** Genetic structure of the WT and IDE-KO alleles. The WT allele produces both exonic and intronic amplicons, while the IDE-KO allele only generates the intronic amplicon, producing a shorter fragment than the WT one due to removal of exon 3. A representative genotyping result is shown (exonic amplicons in the upper part of the gel; intronic amplicons in the lower).

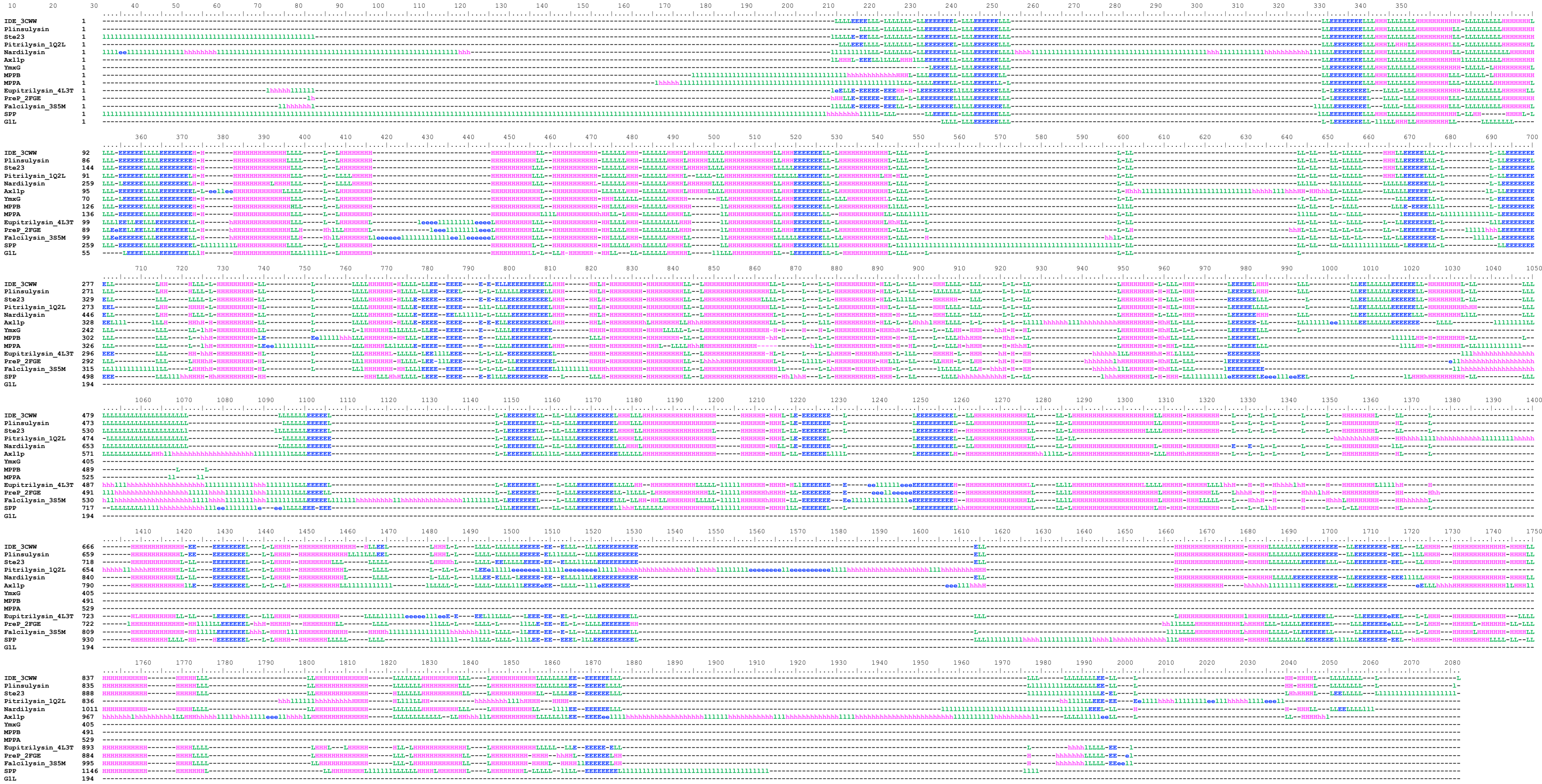

Figure S2. Structural alignments of clan ME metalloendopeptidases. Structural assignments by DSSP, mapped on an underlying sequence alignment, are shown as follows: helix (H/h, in pink), strand (E/e, in blue) and coil (L/l, in green). Uppercase means structurally equivalent positions with human IDE (3CWW) sequence. Lowercase means insertions relative to 3CWW.

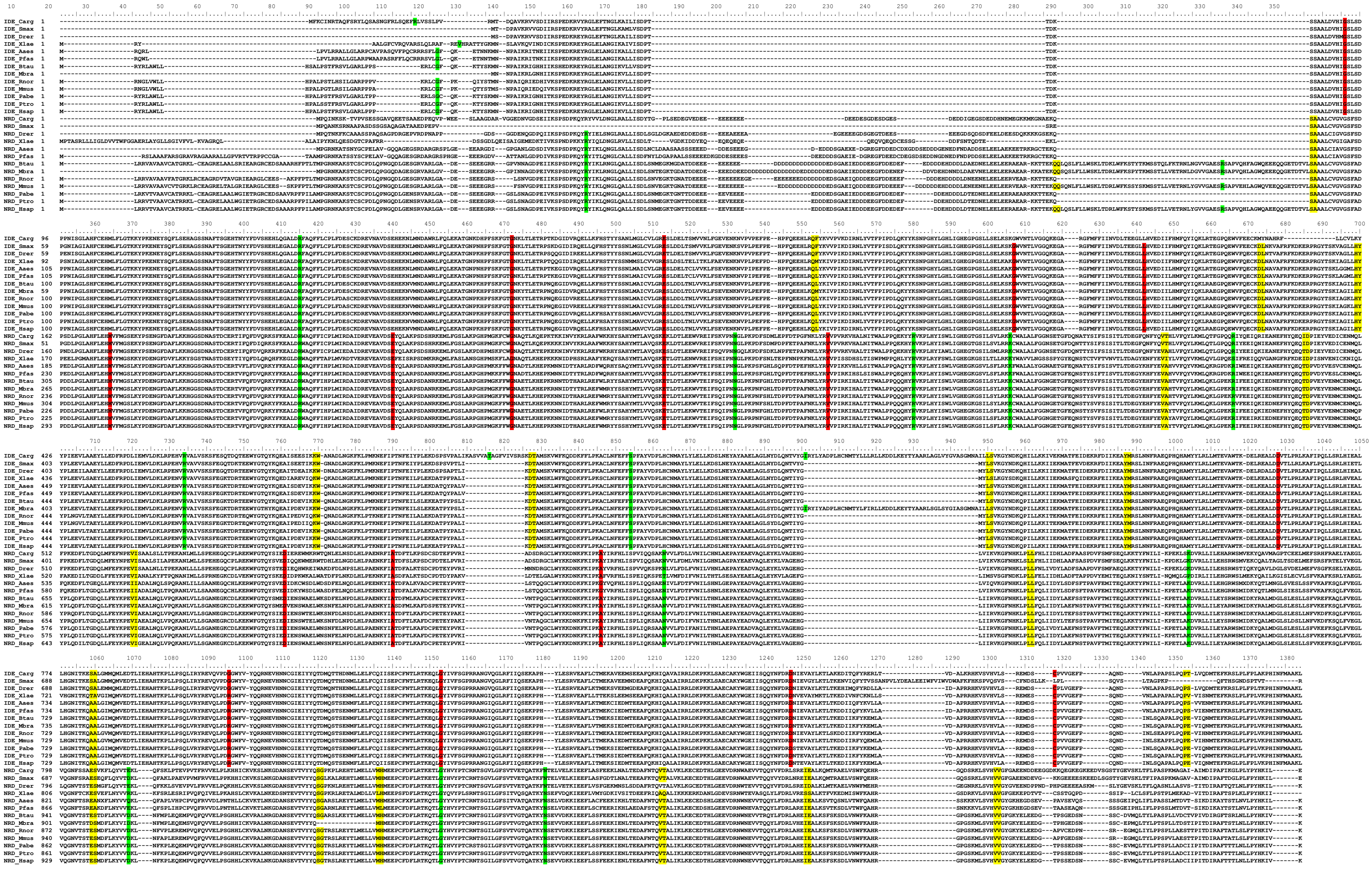

Figure S3. Gene architecture comparison between 26 IDE and NRD proteins. The sequences were retrieved from 13 species of chordates. Introns and their phase (0 = yellow, 1 = red, 2 = green) are depicted on a MSA of protein sequences.

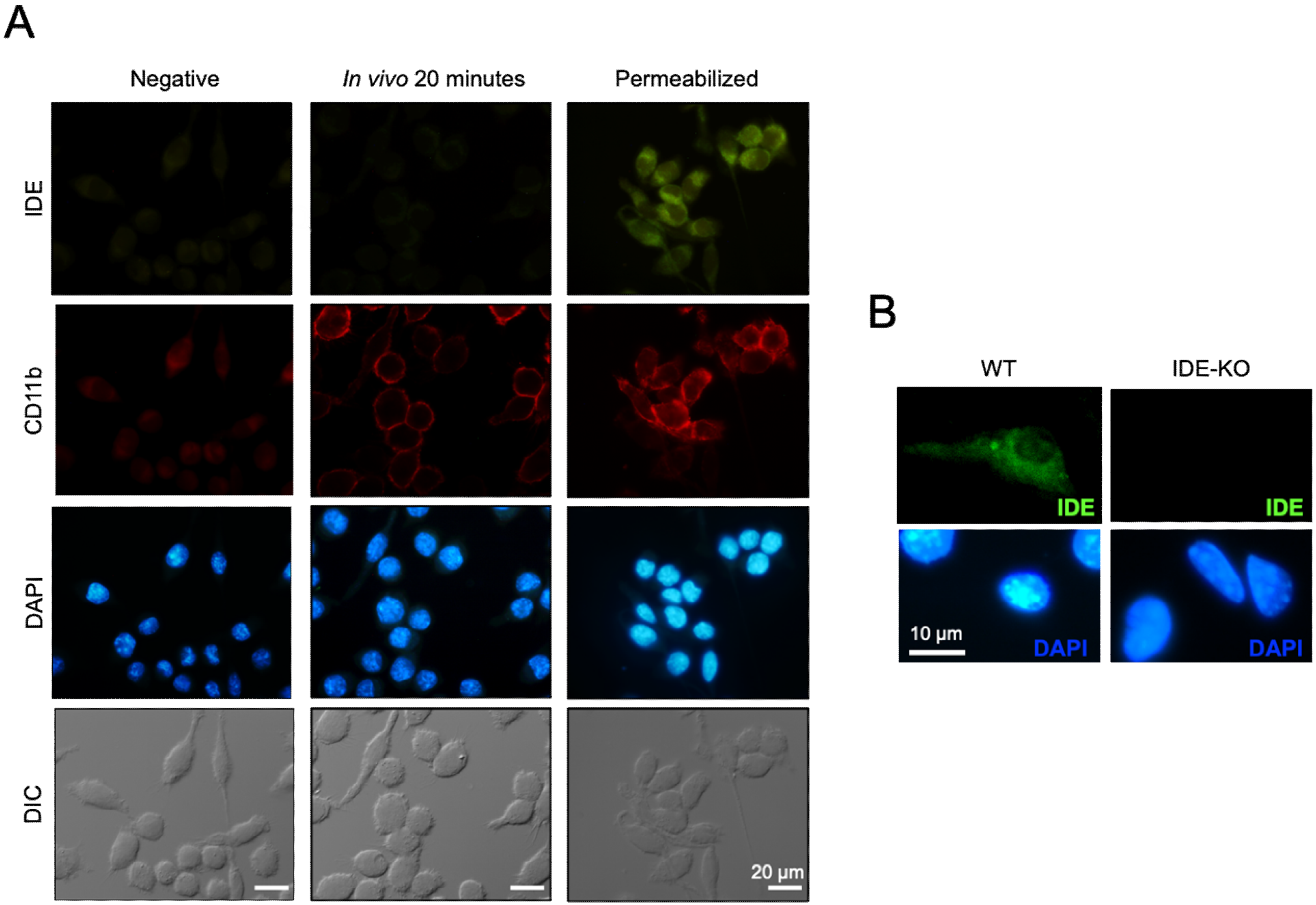

Figure S4. Controls for microscopy experiments. A) Double immunolabeling of IDE and CD11b in BV-2 microglial cell line. Negative control: All steps performed, but no primary antibody added. **B)** Validation of the labeling protocol with anti-IDE antibody in WT and IDE-KO microglial cells.

Molecular phylogeny data S2. Trees output (text format)

Figure 1A

(Eupitrilysin_Hsap:1.0191103455,(PreP1_Atha:0.7225082131,Falcilysin_Pfal:1.3415617952)84:0.2457498546,((MPPA_Hsap:1.0363378193,(MPPB_Hsap:0.71954800
79,YmxG_Rpro:1.2491304481)61:0.2537924513)100:1.0639391740,(((((NRDC_Hsap:1.0268046682,((Ste23_Scer:0.8832602370,IDE_Hsap:0.5509579495)94:0.17568
42058,PIinsulysin_Slyc:0.7650341070)67:0.1260218911)89:0.2364566254,Pitrilysin_Ecol:1.3800937785)67:0.2110329985,Axl1p_Scer:1.7584369669)87:0.40378584
39,G1L_MP44_Vvir:2.8498386663)82:0.3549218325,SPP_Atha:1.8855216901)71:0.1808566439)100:1.1295031830);

Figure 1B

(((((((3CWWA:9.1,s005A:9.6):7.95,Ste23:18.05):3.51666666666666,1Q2LA:22.8666666666667):5.38333333333333,NRD:27.15):4.21,Axl1p:30.66):5.5566666666666
7,(((MPPB:19.2,s008A:21.1):5.3,MPPA:17.3):15.5333333333333,SPP:38.3333333333333):0.68333333333333):5.23333333333333,((4L3TA:23.6,2fgeA:25.1):3.05,3S5
MA:21.85):15.8):4.38846153846154,G1L:20.1384615384615);

Figure 2A

(IDE_BosTau:0.0051226676,((IDE1_HomSap:0.0034152175,((IDE_MusMus:0.0313582878,(((((IDE_AsaScu:0.0034655968,((((IDE_AegBen:0.0026783498,IDE_CeuAer:
0.0053617161)94:0.0000023284,(IDE_ChlAen:0.0008853988,(IDE_ArdKor:0.0017674945,IDE_EryMcc:0.0103529279)90:0.0014782163)60:0.0000027158)78:0.00131
75698,IDE_PatFas:0.0074236233)83:0.0018153438,IDE_CrySou:0.0026377567)91:0.0049848728)100:0.0100945348,IDEa_AllMis:0.0200561702)79:0.0065752659,ID
E_PlaMeg:0.0636724574)100:0.0105380221,IDE_VarKom:0.0626664241)97:0.0114959209,(IDE_XenTro:0.0557440240,(((IDE_AciRut:0.0283482189,(IDE_DanRer:0.0
309783686,(IDE_OryMel:0.0454762402,IDE_ScoMax:0.0619811144)100:0.0164920130)100:0.0162039894)100:0.0149729204,IDE_PolSen:0.0838444480)100:0.024
0385862,(hIDE_ChiPun:0.0989612859,((((((((IDE_MytCor:0.3048386029,IDEl_DimGyr:0.4106404761)94:0.0580096281,(IDEl_OctVul:0.4255844946,IDE_HalDiv:0.490
4052137)80:0.0317921172)86:0.0537259923,((((IDE_TriPse:0.5118839273,(IDE_ToxCan:0.3444612522,(IDEl_AngCos:0.2258438388,M16_HaeCon:0.0716814338)10
0:0.3316412166)100:0.3335359656)100:0.1541703330,(((((((((((((Ste23_ZymBre:0.2636082991,LuxS_PolCit:0.2806531450)100:0.0753690878,Ste23l_DelStr:0.2405
846164)100:0.0975453823,M16_MacPha:0.1856175074)99:0.0289537873,FLC2_VenNas:0.2571988862)100:0.0651601036,(Ste23_LasPus:0.2316374122,(MEN_Pen
Cam:0.1907073750,IDE_BlaDer:0.2406610851)100:0.0859125499)99:0.0358979135)82:0.0322294401,((pMUG_GolCic:0.2012652891,LuxS_AceMac:0.1421937153)
100:0.0660006894,((IDE_FusCir:0.1925498958,ZNP_ColTru:0.1041068861)100:0.0457397370,Ste23_NeuCra:0.2566786688)100:0.0796120041)100:0.1183254583)1
00:0.0938286948,MEN_TryHyb:0.3433919129)97:0.0521271477,NRD_ArtEnt:0.5232627774)100:0.1811330241,IDE_WicCif:0.7216300887)70:0.0533710211,(MEN_
ProLac:0.5729010753,MTP_SchCry:0.7335138471)83:0.0901832623)80:0.0551253095,((((MTE_JimFla:0.1700590519,(IDE_ChoCuc:0.1035073466,UCC_PhyBla:0.136
8645469)100:0.0992350831)100:0.0683114548,((MTE_AbsRep:0.2481554085,Ste23_LicCor:0.1351417306)99:0.0563981733,IDE_ApoOss:0.2099403183)100:0.199
4638424)100:0.0882951418,(MTE_GigMar:0.2502909208,Ste23_RhiIrr:0.2072019057)100:0.1126151584)100:0.1387023266,((Ste23_MalPac:0.4366355957,NRD1_
MoeAnt:0.3629373661)100:0.1389553296,((((LuxS_LeuCre:0.1825182270,IDE_RhoMuc:0.2504483501)100:0.2467553086,IDE_PucGra:0.6287050649)100:0.083771
6825,IDE_WalIch:0.6526696218)97:0.0521433822,((IDE_CryDep:0.3020285088,AFA_TriAsa:0.3669052913)100:0.3515443791,(((Ste23_MonRor:0.3043459295,IDE_
MycKen:0.3890260539)100:0.2301031385,LuxS_GauMor:0.4306182631)100:0.0898043556,IDE_DacPri:0.6183727108)100:0.2098542816)100:0.0836742039)98:0.0
225659292)100:0.0853257642)83:0.0536975162)99:0.1109425148,((LuxS_GonPro:0.7415897449,IDE_RhiGlo:0.6823956191)98:0.0564923681,Ste23_NeoCal:0.967
3084189)98:0.0699958548)100:0.1815729746,((((ZNP_RapSub:0.7049045752,IDEl_ChlPri:0.6685418142)99:0.1128078234,((pIDE_ZosMar:0.2465531509,(ZNP_Apo
She:0.2047536212,((((IDEf_ArtAnn:0.1734506251,((IDEl_OleEur:0.0750912173,IDE_StrAsi:0.1440737846)99:0.0336077248,(IDEl_NicAtt:0.1245654407,Unn_CofCan:
0.2653481687)98:0.0163901225)99:0.0228459232)99:0.0176323222,IDEl_ActChi:0.1021235446)100:0.0375081444,((ZNP_TriWil:0.1073767710,IDE_TheCac:0.0849

450149)96:0.0157788316,(((PQQ_TreOri:0.1014050513,ZNP_PyrUss:0.1350511488)98:0.0218300852,(IDEl_CucMel:0.1801960020,IDEf_AraTha:0.2914679730)86:0.
0229846502)73:0.0055626689,(ZNP_GlySoj:0.1611849710,ZNP_MorRub:0.0934222868)87:0.0194663974)90:0.0102754064)99:0.0266241984)100:0.0331768692,(
M16_MacCor:0.1063889237,M16_CinMic:0.1867351904)98:0.0255475139)100:0.0514740206)100:0.0336330655)100:0.0677137972,ZNP_CarLit:0.1927486289)10
0:0.4276682545)100:0.2640076833,((((Unn_VitBra:0.5612820922,IDE_FisSol:0.6602956553)100:0.2951627207,M16_EmiHux:1.0467366039)98:0.1429274278,(SCG
5_TryCru:0.4663741098,IDE_StrCul:0.4277513511)100:1.3179111860)93:0.1234974721,(((((((((M16_EurArc:0.7311566389,IDEf_HahCcb:0.5201519002)100:0.10926
84329,(IDEf_ParF74:0.7121022762,IDEf_PseHs1:0.7058773128)83:0.1385632332)74:0.0748062301,M16_AciBac:0.7949596430)58:0.0670198277,((IDEf_MetSed:0.1
859299784,M16_MetSp:0.1951217040)100:0.4865370832,M16_AmpJap:0.7200201209)77:0.1247849107)53:0.0307603435,M16_SpoSp:0.9690011125)91:0.09829
93486,(M16_RhoBac:0.9951106902,((IDEf_AerVer:0.0401617534,IDEf_EscCol:0.0404941517)100:0.3950161870,IDEf_PhoPro:0.4147126321)100:0.4357120485)86:0
.1111373506)100:0.2541901383,((((IDEf_LenBac:0.6919858582,(IDEf_BurBac:0.5131657699,IDEf_EreLut:0.3913489649)100:0.2276595411)100:0.1392898668,IDEf_
PlaBac:0.8199349111)100:0.2369700598,(IDEf_Sar324:0.5771000350,M16_DelBac:0.9539942461)74:0.1456334305)100:0.1927851860,((M16_ChlBac:0.462570651
3,PR3_ChlBac:0.3920691262)98:0.1203595999,IDE_ParAca:0.3877574225)100:0.9230023459)97:0.1448330849)91:0.0855100103,(((((IDE_TetThe:0.8598935173,Lux
S_PsePer:0.6517300834)75:0.0988470956,Unn_ParTet:0.7436487685)99:0.1806733886,IDE_StyLem:1.0750269274)100:0.2099458259,(((IDE_MimVir:0.6530758533
,ZNP_HomVir:0.6590510677)97:0.1173967882,ZNP_IndVir:0.8759103648)100:0.3513790410,IDE_YasVir:1.1437080081)100:0.2841438849)80:0.1190722389,(((TLN
1_BesBes:0.1437497284,M16_NeoCan:0.1418117397)100:0.9206333955,((IDE_EimBru:0.0450761344,SDP_EimBov:0.0837449309)100:1.4453643490,IDEl_CryUbi:1.
2952873699)78:0.1597229290)73:0.0747446714,IDE_CavFas:1.2535077945)79:0.1100223603)97:0.1020066718)96:0.0771555038,(IDE_PlaFun:0.9837451678,((((((((
(((((NRD_CamDro:0.0316893968,NRD2_LynPar:0.0130652211)100:0.0021151166,NRD_RhiFer:0.0124975531)100:0.0044022032,NRDb_HomSap:0.0209530555)99:
0.0067473174,NRD1_MusMus:0.0291652791)100:0.0944518860,((((NRD_PatFas:0.0268517849,NRD_LepDis:0.0112978142)100:0.0068812544,NRD_LonStr:0.0453
960813)66:0.0057377716,NRD_GalGal:0.0298394151)100:0.0311004864,(NRD_AllMis:0.0306384839,RhoG_PlaMeg:0.0377130677)100:0.0141987692)100:0.10561
27282)100:0.0914859042,NRD_XenLae:0.3936035895)71:0.0254997998,hNRD_ChiPun:0.2391344620)73:0.0450646577,((NRD_NotFur:0.0632070864,NRD_ColLuc:
0.0585101020)100:0.1197757228,((NRD_SclFor:0.0352350585,NRD_PolSen:0.2645028203)100:0.0403579785,NRDb_DanRer:0.2206498534)93:0.0271377043)95:0
.0401397630)100:0.2951075589,(NRD1_BugNer:0.5663014041,NRDl_DimGyr:0.7013227566)67:0.1230932106)36:0.0416530461,(NRDl_FraOcc:0.6206292773,NR
D_PenVan:0.5089705391)100:0.2086979909)94:0.0822232253,NRD_PhaMam:0.8009536657)100:0.2759542798,(((((MMS19_PhyNic:0.0673762565,NRD_PhyPal:0.
0558328535)100:0.3661782536,NRD_ThrCla:0.5230668087)100:0.4141157080,NRD_NanGad:0.9473360677)99:0.1188289711,(((LuxS_DunSal:0.4569396120,NRDl
_ScePab:0.4128745854)100:0.1294165072,IDE_TreA12:0.3704535032)100:0.3170276870,((NRD_CocNuc:0.1330166279,IDE_AnaCom:0.1283853082)100:0.081243
7054,(IDE_ThaTha:0.1702442660,((NRD_SalSpl:0.1325880807,NRDl_DorHyg:0.1612904993)100:0.0853565879,((NRD_PopAlb:0.1434608017,IDE_MorNot:0.13524
35404)81:0.0236158553,M16_CepFol:0.1516805645)100:0.0396004382)100:0.0923959918)84:0.0291052159)100:0.5339373245)100:0.1203256466)98:0.10616763
62,Ste23_ChrTob:1.1412515215)23:0.0361023962)59:0.0413755848,(pUnn_MonBre:1.0222720014,NRD_SymMic:1.0407150920)73:0.1784336278)100:0.15359618
10)91:0.0948116048)58:0.0279496915)86:0.0302141331)35:0.0388533246,((PEP_AcaCas:0.8315718852,(((((pIDE_AlbCan:0.5899687093,M16_PhyInf:0.2659754606
)100:0.1745354757,M16A_ThrCla:0.4782232478)100:0.1181553665,IDE_PhyNic:0.7167402676)100:0.1742638415,IDE_HonFer:0.7615154022)100:0.0761179662,M
16_FraCyl:0.9664542316)70:0.0499773267)61:0.0422480655,(((IDE_PorPur:0.5634884803,IDE_GraCho:0.7612337627)100:0.1744180192,IDE_GalSul:1.0155876839)
94:0.1048641132,(IDE_ChrTob:0.8130553830,(((IDE_SymMic:0.8247152460,PR3_FisSol:0.9375198207)91:0.0753722524,IDEl_EctSil:0.5966752415)96:0.081960094
7,IDE_NanGad:0.5883543955)100:0.1989019565)99:0.1146092033)58:0.0534097644)38:0.0507221017)94:0.0692598419)100:0.1005150527,(IDE_EchGra:0.692365
6109,(M16_OpiViv:0.3180740671,IDE_FasHep:0.2274878023)100:0.3499346043)100:0.3266503389)100:0.0757560612)100:0.0657598089,IDE_BugNer:0.66569082
32)87:0.0355440269,(IDE_HypDuj:0.6169115849,IDE_BraPli:0.7040571493)78:0.0990216891)68:0.0332104934)69:0.0155027561,IDE_ArgBru:0.4778894759)67:0.0
108396707,(((IDEl_DapMag:0.4143013192,(IDE_ArmVul:0.2490647563,M16_TriLon:0.4124610621)100:0.1321984538)87:0.0493989981,((((IDE_ZooNev:0.1265308
793,IDEl_TimBar:0.1851098776)100:0.0719029170,(IDE_TriCas:0.2750326810,IDE_OocBir:0.4147839673)100:0.0726823343)99:0.0529233415,IDEp_PedHum:0.512

4789594)97:0.0540882428,IDE_PapXut:0.5977617159)66:0.0515961024)63:0.0470704695,(IDE_TigJap:0.5370540392,(((IDE_DroMel:0.5536639849,IDEX1_AphCra:
0.6441393840)70:0.0515924443,IDE_OrcCin:0.6305270096)67:0.0542715637,IDEl_DarSte:0.5482961125)63:0.0389488606)63:0.0395488930)76:0.0632258547)100
:0.0725781289,IDEp_ApoJap:0.4023461561)88:0.0349000105,IDE_PhaMam:0.4867649760)90:0.0394435022,IDE_ExaDia:0.4183929176)100:0.1586562018)100:0.0
361790093)100:0.0327046975)100:0.0237141628)100:0.0310299860)100:0.0079273644,IDE_TupChi:0.0234432889)87:0.0021787377)88:0.0008668446,IDE_PteAle
:0.0137141318)88:0.0008547083,IDE_CanLup:0.0059361587);

Figure 2C

(IDE_Ptro:0.0010037341,IDE_Hsap:0.0000020519,((IDE_Rnor:0.0053803549,IDE_Mmus:0.0077751287)100:0.0364460367,(IDE_Xlae:0.0600759991,(IDE_Drer:0.0558410017,(IDE_Pmam:0.4529526827,((IDE_Dmel:0.6278166956,IDE_Cele:0.9864985139)58:0.1276774261,((S23p_Scer:0.9721498743,IDE_Atha:1.0959203020)39:0.0941957331,((M16_Earc:1.0361309585,Pit_Ecol:1.7984595718)99:0.4331067189,IDE_Tthe:1.3876230862)62:0.2059447514)65:0.1389267330)98:0.1495937229)100:0.3299282783)95:0.0396513328)100:0.0506563962)84:0.0078443214);

Figure 3A

(IDE_Carg:0.0560928078,IDE_Smax:0.0940596680,(IDE_Drer:0.0340974777,((IDE_Xlae:0.0702827827,((((NRD_Carg:0.0695766729,NRD_Smax:0.0655395338)100:0.1543387321,NRD_Drer:0.1077816996)98:0.2002996826,((NRD_Aaes:0.0522437226,NRD_Pfas:0.0306480503)100:0.1999932787,(((NRD_Btau:0.0242722775,NRD_Mbra:0.0367874729)80:0.0129985688,(NRD_Rnor:0.0158058973,NRD_Mmus:0.0084409695)100:0.0440444788)155:0.0056752454,(NRD_Pabe:0.0075305581,(NRD_Ptro:0.0009368834,NRD_Hsap:0.0008994022)91:0.0017949972)99:0.0203317977)99:0.1087087770)98:0.1008993581)95:0.2327388422,NRD_Xlae:0.3944081518)100:3.1360224120)53:0.0162582345,((IDE_Aaes:0.0057137672,IDE_Pfas:0.0089186239)100:0.0383130744,(IDE_Btau:0.0064440043,((IDE_Mbra:0.0143381106,(IDE_Pabe:0.0072519099,(IDE_Ptro:0.0012044661,IDE_Hsap:0.0000027819)68:0.0000027820)71:0.0058399812)35:0.0014001227,(IDE_Rnor:0.0072539338,IDE_Mmus:0.0084809501)99:0.0469688014)29:0.0015418635)63:0.0482373180)62:0.0296454358)100:0.0781553957)93:0.0247770105);
